# Identification and characterization of miRNAs in ripening fruit of *Lycium barbarum* L. using high-throughput sequencing

**DOI:** 10.3389/fpls.2015.00778

**Published:** 2015-09-25

**Authors:** Shaohua Zeng, Yongliang Liu, Lizhu Pan, Alice Hayward, Ying Wang

**Affiliations:** ^1^Key Laboratory of Plant Resources Conservation and Sustainable Utilization, South China Botanical Garden, Chinese Academy of SciencesGuangzhou, China; ^2^Guangdong Provincial Key Laboratory of Applied Botany, South China Botanical Garden, Chinese Academy of SciencesGuangzhou, China; ^3^Queensland Alliance for Agriculture and Food Innovation, The University of QueenslandSt Lucia, QLD, Australia

**Keywords:** fruit ripening, high-throughput sequencing, *Lycium barbarum* L., miRNA, pigmentation

## Abstract

MicroRNAs (miRNAs) are master regulators of gene activity documented to play central roles in fruit ripening in model plant species, yet little is known of their roles in *Lycium barbarum* L. fruits. In this study, miRNA levels in *L. barbarum* fruit samples at four developmental stages, were assayed using Illumina HiSeqTM2000. This revealed the presence of 50 novel miRNAs and 38 known miRNAs in *L. barbarum* fruits. Of the novel miRNAs, 36 were specific to *L. barbarum* fruits compared with *L. chinense*. A number of stage-specific miRNAs were identified and GO terms were assigned to 194 unigenes targeted by miRNAs. The majority of GO terms of unigenes targeted by differentially expressed miRNAs are “intracellular organelle,” “binding,” “metabolic process,” “pigmentation,” and “biological regulation.” Enriched KEGG analysis indicated that nucleotide excision repair and ubiquitin mediated proteolysis were over-represented during the initial stage of ripening, with ABC transporters and sulfur metabolism pathways active during the middle stages and ABC transporters and spliceosome enriched in the final stages of ripening. Several miRNAs and their targets serving as potential regulators in *L. barbarum* fruit ripening were identified using quantitative reverse transcription polymerase chain reaction. The miRNA-target interactions were predicted for *L. barbarum* ripening regulators including miR156/157 with *LbCNR* and *LbWRKY8*, and miR171 with *LbGRAS*. Additionally, regulatory interactions potentially controlling fruit quality and nutritional value via sugar and secondary metabolite accumulation were identified. These include miR156 targeting of fructokinase and 1-deoxy-D-xylulose-5-phosphate synthase and miR164 targeting of beta-fructofuranosidase. In sum, valuable information revealed by small RNA sequencing in this study will provide a solid foundation for uncovering the miRNA-mediated mechanism of fruit ripening and quality in this nutritional food.

## Introduction

Small RNAs of 18–30 nucleotides (nt) guide regulatory processes at both the DNA and the RNA level within organisms. In most cases, ~21 nt long plant microRNAs (miRNAs) are processed from single-stranded small RNAs digested successively by DICER-LIKE1 (DCL1) enzymes in two stages (Chen, 2009), finally resulting in the biogenesis of a mature miRNA duplex. This duplex is methylated at the 3′ end by HEN1 and transported into the cytoplasm (Yang et al., 2006). One strand of the duplex, known as the guide-strand (miRNA), is integrated into AGRONAUT (AGO) proteins to form an RNA-induced silencing complex (RISC; Khvorova et al., 2003; Schwarz et al., 2003). while the passenger-strand (miRNA^*^) of the duplex is usually degraded. The mature miRNA-RISC complex is what mediates downstream regulatory processes, either by inducing cleavage or translational repression, of complementary transcripts. Plant miRNAs have been experimentally analyzed and bioinformatically predicted in many species, including pear (*Pyrus bretschneideri* Rehd.; Wu et al., 2014), orange (*Citrus sinensis* [L.] Osbeck; Liu et al., 2014a), and tomato (*Solanum lycopersicum* L.; Mohorianu et al., 2011). Such studies revealed miRNAs to be master regulators, targeting transcription factors (TFs) involved in diverse physiological processes including fruit ripening (Mohorianu et al., 2011; Ferreira e Silva et al., 2014; Chen et al., 2015).

A number of miRNAs serve as master regulators of fruit ripening via mRNA cleavage and/or translational repression of ripening-related TFs. The miRNAs miR156/miR157 and miR172 function in a linear pathway to orchestrate vegetative and reproductive transitions (Chuck et al., 2007). miR156/miR157 regulates the SQUAMOSA PROMOTER BINDING PROTEIN-LIKE (SPL) TFs, including fruit-ripening regulator tomato *SlCNR* (Ferreira e Silva et al., 2014), in species including persimmon (*Diospyros kaki* Thunb; Luo et al., 2015), Arabidopsis [*Arabidopsis thaliana* (L.) Heynh] (Xie et al., 2005), and pear (Wu et al., 2014). In tomato, miR157 controls *SlCNR* in a dose-dependent manner through both mRNA cleavage and translational repression (Chen et al., 2015). Noticeably, previous studies show that the *cnr* mutation has profound effects on ripening-related gene expression (Eriksson et al., 2004) and carotenoid biosynthesis (Fraser et al., 2001) in tomato fruit. Meanwhile, miR172 regulates the tomato ERF TF *SlAP2a*, which is a negative regulator of ripening (Chung et al., 2010; Karlova et al., 2013). The miRNA, miR169, suppresses C class MADS box genes in relation in carpel development by inhibiting the expression of NF-YA TFs (Cartolano et al., 2007), suggesting that miR169 is involved in fruit development. miR164 targets NAM/ATAF/CUC (NAC) TF family members (Mallory et al., 2004; Guo et al., 2005; Nikovics et al., 2006), including *SlNAC4*, a positive regulator of fruit ripening in tomato (Zhu et al., 2014). The role of the plant hormone ethylene in fruit ripening is well established. Recently, ethylene was shown to regulate miRNAs, including miR156, miR390, miR396, and miR4376 during fruit ripening in tomato (Gao et al., 2015). This regulation is dependent on Ripening INhibitor (RIN), which serves as master regulator of fruit ripening by controlling ripening-related TFs such as CNR, AP2, and Non-ripening (NOR) (Fujisawa et al., 2012, 2013), as well as the above miRNAs and miR172 (Gao et al., 2015).

The Solanaceae species *Lycium barbarum* L. has been extensively utilized as a traditional medicinal plant in China for thousands of years (Potterat, 2010). This is attributed to a great extent to the high level of health-promoting bioactive components including polysaccharides, flavonoids, and carotenoids in *L. barbarum* fruits (Potterat, 2010; Amagase and Farnsworth, 2011). *L. barbarum* fruit extracts have antitumor, immune enhancing, hepatoprotective, and neuroprotective properties (Amagase and Farnsworth, 2011). Our recent work reveals that the content of bioactive carotenoids, in *L. barbarum* fruits, is enhanced during fruit ripening, reaching maximum levels in ripe fruit (Liu et al., 2014b). This suggests that fruit ripening might modulate the accumulation of bioactive components, at least for carotenoids. As described above, multiple miRNAs participate in controlling fruit ripening. High-throughput small RNA sequencing is a time-saving and cost-effective approach to identify miRNAs involved in biological processes. To date, a large number of miRNAs in fruit are identified in plants including tomato (Gao et al., 2015), pear (Wu et al., 2014), persimmon (Luo et al., 2015), and orange (Liu et al., 2014a). Recently, miRNAs were characterized using high-throughput sequencing in fruits and shoot tips of *Lycium chinense* P. Mill. (Khaldun et al., 2015), which is the closest relative of *L. barbarum* in the genus *Lycium* (Levin and Miller, 2005). However, that study focused on identifying the tissue-specific miRNAs with less attention paid to ripening-related miRNA in fruits. So far, miRNAs have not been identified in *L. barbarum* fruits, and very little is known about *Lycium* miRNAs governing fruit ripening in the two related species.

In this study, four fruit samples covering four developmental stages (S1–S4) of *L. barbarum* fruit ripening, were sequenced using an Illumina HiSeq™ 2000 platform. Bioinformatic analysis revealed 38 known and 50 novel miRNAs in *L. barbarum* fruits with stage-specific miRNAs in each of S1, S2, S3, and S4. Target gene prediction and GO annotation revealed 194 putative target genes of miRNAs. Furthermore, enriched GO and KEGG analysis of differentially expressed miRNAs was performed to begin to uncover the miRNA-mediated mechanism of fruit ripening. Quantitative reverse-transcription polymerase chain reaction (qRT-PCR) was adopted to validate the expression level of miRNAs and their target genes in ripening fruits. Noticeably, several candidate genes potentially controlling fruit ripening and fruit quality were identified and discussed in this study.

## Materials and methods

### Plant materials

*L. barbarum* L. fruits at four developmental stages were harvested from Zhongning County, Ningxia Hui Autonomous Region, China. These stages were: S1 stage (green fruit, 3 days before color break), S2 stage (the color-break stage), S3 stage (light-red, 3 days after color break), and S4 stages (ripe red fruit, 6 days after color break). For small RNA sequencing, each fruit sample was collected from more than three independent individuals and pooled together. For validation of gene expression, fruit from three independent biological replicates per stage were analyzed. All samples were immediately frozen in liquid nitrogen after harvest, and stored at −80°C until further use.

### RNA isolation, small RNA library construction, and sequencing

Total RNAs were isolated using TRIzol (Invitrogen, USA) according to the manufacturer's instructions. RNA purity and integrity were evaluated using agarose gel electrophoresis and an Agilent bioanalyzer 2100 (Agilent, USA) and quantified by a Qubit 2.0 Fluorometer (Life Technology, USA). Five microgram of high quality total RNA was used to construct each small RNA library and sequenced using a HiSeq™ 2000 at the Novogene Company, Beijing, China. The small RNA dataset was deposited in the National Center for Biotechnology Information Databank (accession SRP062403).

### Bioinformatic analysis

After sequencing, raw sequences were filtered to remove low quality sequences, adapter sequences, and reads with poly N. The clean reads were blasted against the RepeatMarker and Rfam databases (http://www.sanger.ac.uk/software/Rfam) to exclude known non-coding RNAs, including rRNAs, tRNAs, snRNAs, snoRNAs. Any fragments encoding protein were also discarded by blasting against the reference unigenes derived from a fruit transcriptomic dataset of *L. barbarum* L. (Zeng et al., unpublished data). The remaining sequences were searched against the miRBase19.0 database to identify putative known miRNAs. The final miRNAs dataset was subjected to analysis of length distribution and nucleotide preference at each position. Novel small RNAs not mapping to miRBase were predicted using both miREvo (Wen et al., 2012) and mirdeep2 (Friedländer et al., 2012). Simultaneously, for each candidate novel miRNA, the miRNA count, length and nucleotide bias at each position was calculated.

### Analysis of differentially expressed miRNAs

To define the expression level of miRNAs, miRNA count was normalized as transcripts per million (TPM) with the formula normalized expression = mapped read count/total reads^*^10^6^ (Zhou et al., 2010). Differential expression analysis of two samples was performed using the DEGseq (Wang et al., 2010) R package. *P*-values were adjusted using *q*-value (Storey and Tibshirani, 2003). The criteria of *q* < 0.01 and |log2(foldchange)| > 1 was set as the threshold for defining statistical significant different expression.

### Target gene prediction and enrichment analysis

Putative target genes of miRNAs identified in this study were predicted *in silico* using psRobot software (Wu et al., 2012) based on sequence similarity to the fruit transcriptomic dataset of *L. barbarum* (Zeng et al., unpublished data). Gene Ontology (GO) enrichment analysis was performed on the target gene candidates of differentially expressed miRNAs. GOseq (Young et al., 2010) was implemented for GO enrichment analysis using Wallenius' non-central hyper-geometric distribution method. Enriched GOs were plotted using WEGO (http://wego.genomics.org.cn/cgi-bin/wego/index.pl). KEGG (Kanehisa et al., 2008) is a database resource for understanding high-level functions and utilities of the biological system. In this study, KOBAS software (Mao et al., 2005) was utilized to test the statistical enrichment of the target gene candidates of differentially expressed miRNAs in KEGG pathways.

### Quantitative RT-PCR (qRT-PCR) and stem-loop qRT-PCR

Total RNAs were digested with a gDNA Eraser Kit (TaKaRa, Japan) to remove any remaining genomic DNA according to the manufacturer instructions. Subsequently, 3 μg RNA was reverse-transcribed in a 20 μL reaction mix containing 4.0 μL 5X PrimeScript Buffer 2, 1.0 μL PrimeScript RT Enzyme Mix I, 1.0 μL (10 μM) Gene Specific Primers. Each qRT-PCR reaction was 20 μL, comprising of 10 μL 2X SYBR *Premix Ex* Taq II (Tli RNaseH Plus), 0.8 μL PCR Forward Primer (10 μM), 0.8 μL PCR Reverse Primer (10 μM), and 200 ng template. qRT-PCR was performed using a LightCycler 480 Real-Time System (Roche, Switzerland) as follows: 95°C for 5 min followed by 45 cycles of 95°C for 10 s, 60°C for 30 s, 72°C for 10 s, and melt curve analysis from 65 to 95°C. For target genes, primers used in this study were designed using Primer 5.0 (Table S1). To normalize gene expression, *Actin1* (Zeng et al., 2014) and U6 (Turner et al., 2013) were selected as internal controls for protein-coding genes and miRNAs, respectively. For miRNA expression, stem-loop qRT-PCR primers (Table S1) were designed as previously described (Varkonyi-Gasic and Hellens, 2011). All qRT-PCR reactions were performed with three biological replicates and the relative gene expression level was analyzed using the 2^−ΔΔCt^ method (Livak and Schmittgen, 2001).

## Results

### Small RNA libraries for *Lycium* fruit ripening

**Figure 1:**
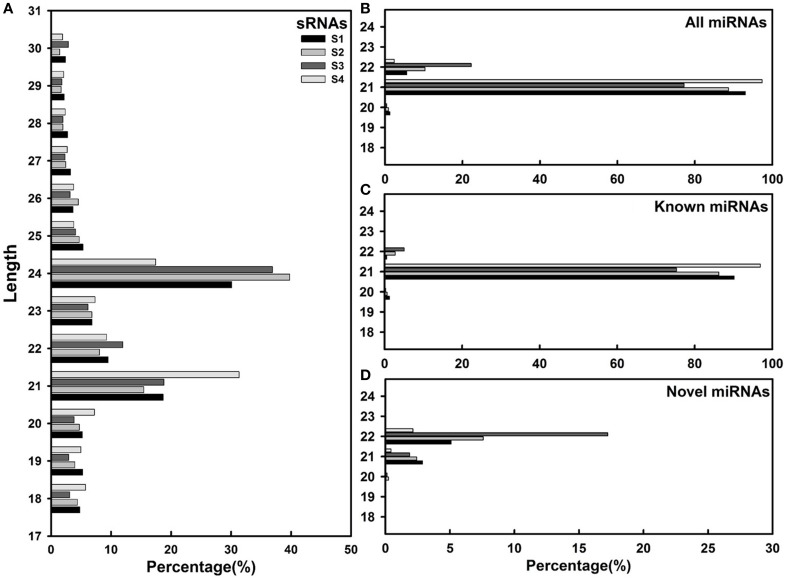
**Length distribution of sRNAs in *Lycium barbarum* L. ripening fruits**. **(A)** sRNAs; **(B)** All miRNAs; **(C)** Known miRNAs; **(D)** Novel miRNAs.

To uncover the regulatory roles of miRNAs in *L. barbarum* fruit ripening, four libraries of small RNAs derived from ripening fruits stages S1-S4 were sequenced using an Illumina HiSeq™ 2000 (Table S2). A total of 8,756,779–13,161,859 raw reads were obtained from the four libraries. After filtering low quality sequences, adapters, and poly Ns, clean reads, ranging from 7,872,479 (for S3) to 11,841,084 (for S4) were obtained. The clean reads were mapped to *L. barbarum* transcriptome data (Zeng et al., unpublished data), resulting in more than 5,570,823 and 1,124,267 raw and unique small RNAs reads in each library, respectively (Table S2). As shown in Figure 1A, in S1–S3 samples, the dominant abundant small RNA length was 24 nt followed by 21, 22, and 23 nt, which is consistent with previous studies in orange (Liu et al., 2014a), pear (Wu et al., 2014), and persimmon (Luo et al., 2015). Dominant small RNA length in stage S4, however, was 21 nt followed by 24, 22, and 23 nt, consisting to that in tomato ripening fruit (Mohorianu et al., 2011). As shown in Figures 1B–D, the abundance of known miRNAs was higher than that of putative novel miRNAs.

In order to globally elucidate the composition and function of small RNAs in each library, small RNA reads were blasted against the miRBase, RepeatMarker, and Rfam databases. This revealed that rRNAs were the most abundant non-coding RNAs followed by repeat, tRNA, snRNA, and snoRNA (Table S3). Noticeably, the abundance of miRNA reads generally increased during fruit ripening with the exception of known miRNAs at S2 and novel miRNA at S4. RepeatMarker identified 63,130 repeats in S1, 40,611 in S2, 33,836 in S3, and 23,255 in S4; with minisatellites and long terminal repeats (LTR) being the dominant repeat small RNA (Figure S1). Pairwise comparisons between stages indicated that a small subset of unique small RNAs shared in two libraries represented the small RNAs with high abundance, and that stage-specific small RNAs seemed to increase with exception of those at stage S4 during fruit ripening (Figure S2).

### Identification of known and novel miRNAs

**Figure 2:**
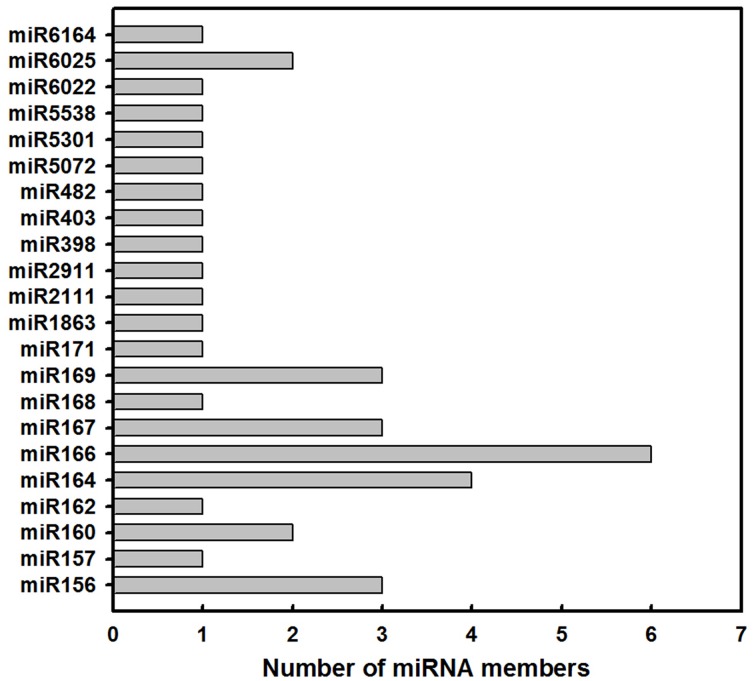
**Summary of the number of miRNA family members in *Lycium barbarum* L. ripening fruits**.

In order to identify known miRNAs in *L. barbarum* fruits, the small RNA reads were used as queries to blast the miRBase database. Consequently, 38 known miRNAs across 22 families were identified (Table 1 and Figure 2). As shown in Figure 2, the number of miRNA members within each family varied from 1 (for miR162) to 6 (for miR166). Furthermore, the miR164, miR156, miR167, miR169, miR160, and miR6025 families consisted of 4, 3, 3, 3, 2, and 2 members.

**Table 1:** **Expression level of known miRNAs identified in *Lycium barbarum* L. ripening fruits**.

**miRNA**	**Sequence (5′–3′)**	**S1**	**S2**	**S3**	**S4**
miR156a	UGACAGAAGAGAGUGAGCAC	20.7	953.8	331.6	17.0
miR156g	CGACAGAAGAGAGUGAGCAC	0.0	0.0	2.0	0.0
miR156j	UGACAGAAGAGAGAGAGCAC	0.0	3.5	0.0	0.7
miR157a	UUGACAGAAGAUAGAGAGCAC	4.6	0.0	3.9	0.0
miR160a	UGCCUGGCUCCCUGUAUGCCA	16.1	14.1	23.4	0.7
miR160e-5p	UGCCUGGCUCCCUGUAUGCCG	16.1	14.1	23.4	0.7
miR162a	UCGAUAAACCUCUGCAUCCAG	1585.3	5330.7	7305.1	626.9
miR164a	UGGAGAAGCAGGGCACGUGCA	549.1	3734.0	5009.2	487.3
miR164c	UGGAGAAGCAGGGCACGUGCG	16.1	109.5	11.7	3.7
miR164d	UGGAGAAGCAGGGCACGUGCU	16.1	109.5	11.7	2.2
miR164e	UGGAGAAGCAGGGCACGUGAG	2.3	28.3	2.0	0.0
miR166a	UCGGACCAGGCUUCAUUCCCC	859659.3	807706.0	698818.7	955364.8
miR166b-5p	GGAAUGUUGUCUGGCUCGGGG	114.9	293.2	216.5	1.5
miR166d-5p	GGAAUGUUGUCUGGCUCGAGG	163.1	303.8	222.4	3.0
miR166e-3p	UCGAACCAGGCUUCAUUCCCC	0.0	3.5	0.0	0.0
miR166g-3p	UCGGACCAGGCUUCAUUCCUC	31096.7	28624.7	27710.5	10762.1
miR166m	UCGGACCAGGCUUCAUUCCCU	3460.0	2370.4	1533.2	782.0
miR167a	UGAAGCUGCCAGCAUGAUCUA	668.6	6005.4	9290.8	305.0
miR167d	UGAAGCUGCCAGCAUGAUCUGG	62.0	512.2	421.3	43.6
miR167d-5p	UGAAGCUGCCAGCAUGAUCUG	55.1	385.1	337.5	42.8
miR168a	UCGCUUGGUGCAGGUCGGGAC	2747.8	3741.0	762.7	289.5
miR169f.1	UAGCCAAGGAUGACUUGCCUA	0.0	0.0	7.8	0.0
miR169h	UAGCCAAGGAUGACUUGCCUG	4.6	0.0	7.8	0.0
miR169t	UAGCCAAGGAUGACUUGCCUU	0.0	0.0	7.8	0.0
miR171a	UGAUUGAGCCGCGCCAAUAUC	4.6	53.0	70.2	0.0
miR1863a	AGCUCUGAUACCAUGUUAGAUUAG	0.0	0.0	0.0	0.7
miR2111a-5p	UAAUCUGCAUCCUGAGGUUUA	0.0	7.1	0.0	0.0
miR2911	GGCCGGGGGACGGACUGGGA	12539.8	5913.6	2775.7	2038.0
miR398b	UGUGUUCUCAGGUCGCCCCUG	195.3	1444.8	838.8	101.9
miR403	UUAGAUUCACGCACAAACUCG	2.3	56.5	15.6	0.0
miR482c	UUUCCUAUUCCACCCAUGCCAA	5403.7	27081.0	50193.3	2549.7
miR5072	CGAUUCCCCAGCGGAGUCGCCA	62.0	35.3	41.0	3.0
miR5301	UGUGGGUGGGGUGGAAAGAUU	1020.1	1854.6	967.5	509.5
miR5538	ACUGAACUCAAUCACUUGCUGC	0.0	0.0	2.0	3.7
miR6022	UGGAAGGGAGAAUAUCCAGGA	4.6	3.5	3.9	0.0
miR6025a	UACCAACAAUUGAGAUAACAUC	0.0	0.0	2.0	0.0
miR6025d	AACAAUUGAGAUAACAUCUAGG	0.0	0.0	2.0	0.0
miR6164a	UCACAUAAAUUGAAACGGAGG	0.0	7.1	5.9	0.0

Expression level of known miRNAs were presented in TPM value (Transcripts Per Million).

To predict novel miRNAs in *L. barbarum* fruits, miREvo combined with mirdeep2 was utilized, resulting in 50 novel miRNAs with stable hairpin structures, designated miRNA01-miRNA50 (Figure S3). To determine the precursor sequences for these putative miRNAs and validate their origin, transcriptome data for the same *L. barbarum* S1–S4 samples was analyzed. Noticeably, the precursors for 32 of the predicted novel miRNAs were discovered. It has been suggested that novel plant *MIR* genes are derived from duplication of protein-coding genes, with a large proportion of Arabidopsis genome-wide novel miRNAs generated in this manner (Cuperus et al., 2011). In this study, three pairs of novel miRNAs; miR03 and miR45, miR05 and miR16, and miR07 and miR17 appear to be processed from the same transcripts corresponding to glutathione S-transferase T1-like, an unnamed protein, and simiate protein, respectively (Data not shown). Of all 50 novel miRNAs, miRNA^*^ of 11 miRNAs were undetectable (Table 2). Apart from miR20^*^, miR22^*^, miR24^*^, miR27^*^, miR46^*^, and miR50^*^, more than two reads for each novel miRNAs^*^ were detected in the four libraries (Table S4), supporting the existence of these novel miRNAs.

**Table 2:** **Expression level of novel miRNAs identified in *Lycium barbarum* L. ripening fruits**.

**miRNA**	**miRNA Sequence (5′–3′)**	**S1**	**S2**	**S3**	**S4**	miRNA^*^ Sequence (5′–3′)	miRNA^*^	**LcmiRNA**
miR01	UUGCCGACUCCACCCAUACCAC	21591.9	31871.2	63553.1	7079.7	GGGGUGGGUGGGGCGGUA	Yes	
miR02	AAACCCUCAGCGAUCCAUAAC	5548.5	3433.7	2436.3	206.0	UUGUGGGUUGCAGGGGGUUUU	Yes	LC35
miR03^a^	UAGGGCGUUCGGAUCCUUCUGC	1808.1	3278.3	4055.3	827.0	AGAAGGAUGCUGACGCCCUUGC	Yes	LC1
miR04	UUACCUGAUCCUGGCAUACCAA	464.1	3235.9	5440.3	62.0	GGCGUGUCAAUAUUGGGUAAGA	Yes	
miR05^b^	AAUCCUUCUGCAAUCCAUAAC	2927.0	2183.2	2543.6	236.3	UGUGGGUUGCAGAGGGUUUUA	Yes	LC11
miR06	UCUUACCAAUACCUCCCAUUCC	546.8	1932.3	3784.2	426.8	AGUGGGAGGUGUUGUAAGAUA	Yes	LC16
miR07^c^	UAGAAAGAGUUUGUAGGCGAG	390.6	1759.2	901.2	208.2	UGCCUACAAACUCUUUCUAUA	Yes	LC14
miR08	GAUCAUGUGGUAGCUUCACC	94.2	2381.0	1377.1	22.9	UGAAGCUGCCAGCAUGAUCUAAAC	Yes	LC4
miR09	UUGCCAAUUCCCCCCAUUCCGA	9906.8	14928.8	26649.3	4115.8	GGAGUGGGUGGCAUGGCAAGA	Yes	LC02
miR10	UUAGAUUCACGCACAAACUUG	204.5	904.3	419.4	25.1	CGUUUGUGCCUGGAUCUGACA	Yes	LC9
miR11	UCCAAUCUCCUCGCCCAUAUUU	576.7	717.1	600.8	89.3	UAUGGAGAGGUGAUUGGAGA	Yes	LC17
miR12	CAUCACAGGUUACUCCAUCCCA	195.3	522.8	842.7	59.8	GGAUGGACUGAAAUGUGAUAAG	Yes	
miR13	GCGAAAGUCGUCUGUGACCCG	11542.7	4776.1	1851.1	437.9	UGUCGCAGGUGACUUUCGCCC	Yes	
miR14	CCCCAUGGACGACCUAAAUACG	500.9	310.9	132.6	11.1	UAUUUAGGUCGACAAGGGGUA	Yes	
miR15	AAUCCCGGGAUUGUAGUGUUAUUU	335.4	381.5	259.4	18.5	CGAUAUAACUAAUCCUGGGAU	Yes	LC33
miR16^b^	AAGCCUUCCGCAAUCCAUAAC	255.0	222.6	156.0	11.8	UGUGGGUUGUAGAGGGUUUUA	Yes	
miR17^c^	UUUUCCCUUGAAUACUCACUU	80.4	137.8	156.0	6.6	UGAGUAUUCAAGGGAAAAUGG	Yes	
miR18	CGCACCCAACAUGGCUCCAUC	0.0	31.8	148.2	3.0	UGGUGCCACGCUGUGUGCGAC	Yes	
miR19	AAUGCUGAAAGAGUCGUGCCU	89.6	45.9	9.8	1.5	CACGACUCUUUCAGCAUUUUU	Yes	
miR20	AAAACCCCCGCGAAUUGCAACUUU	50.5	49.5	21.5	0.7	AGUUGCAAUUCCCGGGAUUUUAGC	Yes	
miR21	CAAUAACAAACUCCAGGAGUG	0.0	28.3	15.6	0.0	CUCCUGUAGUAUUGUUAAUUGG	Yes	
miR22	CCCUUUGAACUGAGAUGUGCCU	57.4	21.2	7.8	2.2	CACUCUCAGUUCACAGAGGCGG	Yes	
miR23	UUUUUGGAGAGUUCGAGCAAC	34.5	17.7	11.7	0.0	UGCUCGGACUCUUCAAAAAAU	Yes	
miR24	GAAUAGGAAAAACAACUU	0.0	53.0	25.4	0.0	CAUUGGUUUCCUGGGCUGUCA	Yes	
miR25	CAAAGGCCACAAGAUUCACUU	0.0	0.0	15.6	0.0	GCAAAUGUUGUGGUCUUAGCA	Yes	LC31
miR26	AAGACAGGGGUAUAUUUGAAAACU	6.9	24.7	25.4	0.7	CAAAUAUACCCGUCUUGGCGA	Yes	
miR27	AUAAUACUUGGAAUAUGCCCU	363.0	823.1	470.1	14.0	CAUAUUCCAAGUAUUAUGGGA	Yes	LC32
miR28	UCGAACCCGUGACCUCAAGCC	18.4	98.9	119.0	40.6	CUUGAGGUUGCGGGUUCGAUA	Yes	
miR29	AUAAACAGACCCUCAAACUUGGCC	18.4	45.9	56.6	0.0	AAGUUUGAGGGCUUGUUUAU	Yes	
miR30	UUAAAAAGGGUUAUGUAGUGGC	50.5	1271.7	524.7	6.6	CACUACAAAGCCCUUCUUAUUU	No	LC3
miR31	UGAUACUGCACUUGGAUACCU	608.8	3465.5	2016.9	344.8	GUAUCCAAGUGCAGUAUCAUA	Yes	
miR32	AUGUAGGAUACUCAGCACUCGCGG	23.0	7.1	9.8	11.8	GGAGGUUGAGUAUCACGCAGUA	No	
miR33	AGCGCCUCCGGGUCCUGUCGACUA	36.8	45.9	37.1	43.6	ACUGAUCCGGUAGGCUGCGCUGCA	Yes	
miR34	UUAUAUGGACUUGGACAAUCC	66.6	21.2	21.5	0.7	AUUGCUCAAGUCCAUUUAAGA	No	
miR35	AAGAGAGUUUCGCUGGGCUUUAGC	11.5	42.4	27.3	3.7	UUCGGCCCAGUAAUUUUUUUCA	No	
miR36	CGAAAUUCGUGCGUGAAAGGACCA	6.9	53.0	19.5	1.5	GCCUUUCACGCAUGAAAUUCGUG	No	
miR37	GCUCACUGCUCUAUCUGUCACC	2.3	21.2	31.2	2.2	UGACAGAAGAGAGUGAGCAC	No	
miR38	CAUAGGAUUCUUGGGCAUGCU	3855.2	2246.7	1410.3	2677.4	AUGUUCAAGAAUCCUAUGCUA	Yes	LC25
miR39	AGGCAUGAGUAUGCCGGGUCCGGC	48.2	38.9	11.7	0.0	CGGACUCAGCAUAUUUAUGCAACG	No	
miR40	UCUUUCCUACGCCUCCCAUACC	15076.2	17638.3	66518.0	8712.3	UGUGGGUGGGGUGGAAAGAUU	Yes	
miR41	ACUUGGGACGAGUUCGUGACUGCU	27.6	0.0	19.5	22.2	CAGACAUGGAUAUUGCCCGACUAC	No	
miR42	UUGCUCGGACUCUUUAAAAAU	4.6	7.1	5.9	0.7	AUUUUUGGAGAGUCCGAGCAAUAU	No	
miR43	AAUGAACCGGACUCCCUUAAG	9.2	0.0	0.0	3.0	UAAGCGAGUCUGGCCCAUUGU	No	
miR44	AGAGGCCUGUAGACAUGUAUG	2.3	14.1	0.0	0.7	GCCAGUCUAGGGUGCCGUUCUGC	Yes	
miR45^a^	UAUGGCGUUCGGAUCCUUCUAC	73.5	60.1	93.6	19.9	GGAUAUCGACGCCCUUGCUCUUA	Yes	
miR46	ACUCCCUCCGUUCACUUUUACUUG	29.9	24.7	25.4	19.2	UAAAAGUGAACGGAGGGAG	Yes	
miR47	CAAAUCCCUCUGCGAUCCAUA	2692.7	4016.6	6109.3	277.6	UGGGUCGCUGAAGGAUUGAUG	Yes	
miR48	UUUUCUACCUGAACUAUCACC	193.0	35.3	13.7	1.5	AUAGUUCAGGUAGAAAAAGGG	No	
miR49	UUAUCUGCCCCUGCCUUUGCAUCC	6.9	10.6	11.7	0.0	AGUAGUUGUAGGUGGUGGAUUAGU	Yes	
miR50	CAAACAAGUUGGGGCCAGCU	75.8	63.6	29.3	3.0	AGCCGACCUCAACUAGUUUAG	Yes	

Expression level of novel miRNAs were presented in TPM value (Transcripts Per Million). miRNAs marked by “a,” “b,” or “c” are processed from the same transcripts. LcmiRNAs column indicated that these novel miRNAs in Lycium chinense were also detected in L. barbarum.

Length distribution analysis showed that the majority of known miRNAs are 21 nt followed by 22 nt, while the majority of novel miRNAs are 22 nt miRNAs (Figures 1B–D). The first cleavage position is critical to determine the mature miRNA sequence and resulting target specificity (Mi et al., 2008; Bologna and Voinnet, 2014). Nucleotide bias analysis indicated that 21–22 nt known miRNAs and 22 nt novel miRNAs preferred U at the first position (Figure S4). Noticeably, the nucleotide bias in known miRNAs was larger than novel miRNAs (Figure S5). During fruit ripening, the nucleotide bias at each position in novel miRNAs fluctuated more than in known miRNAs, especially in the 1st, 10th, and 11th positions (Figures S4, S5). Previous studies suggest that the 1st nucleotide is very important to miRNA sorting (Mi et al., 2008), and that 10th and 11th nucleotides are responsible for guiding miRNA to cleave target mRNA (Martinez and Tuschl, 2004). Taken together, these results suggest that novel miRNAs might be involved in regulating physiological or biological processes distinct to those controlled by known miRNAs in *L. barbarum* fruits.

**Figure 3:**
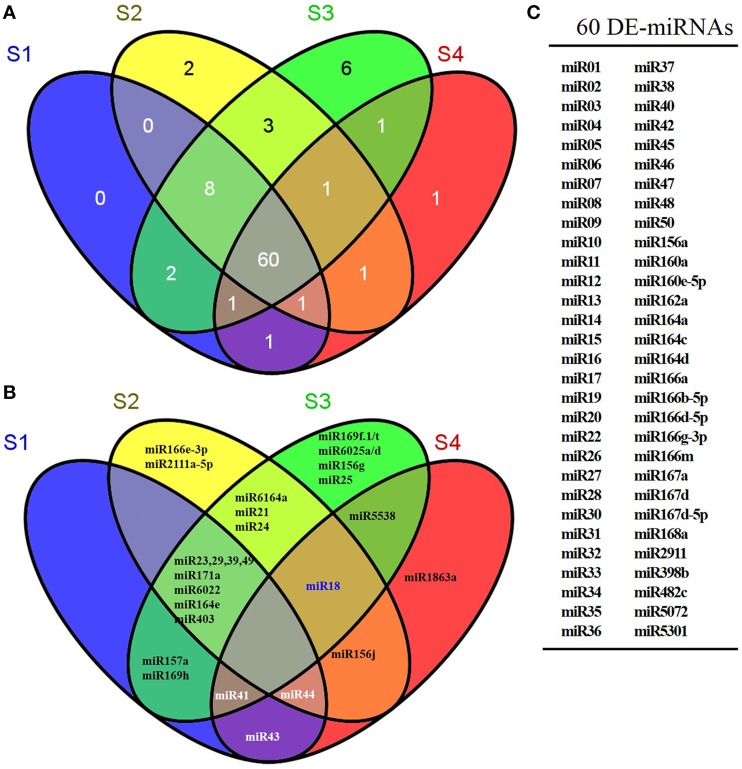
**Venn distribution of differentially expressed miRNAs in ripening fruit samples**. **(A)** showed the number of differentially expressed miRNA in ripening fruits. **(B)** showed the exact miRNA(s) differentially expressed in ripening fruits. **(C)** indicated that the 60 miRNAs differentially expressed in all ripening fruit samples. The differentially expressed *Lycium barbarum* miRNAs were defined as following the criteria of *q* < 0.01 and |log2(foldchange)| > 1 based on the number of miRNA reads in small RNA libraries.

**Figure 4:**
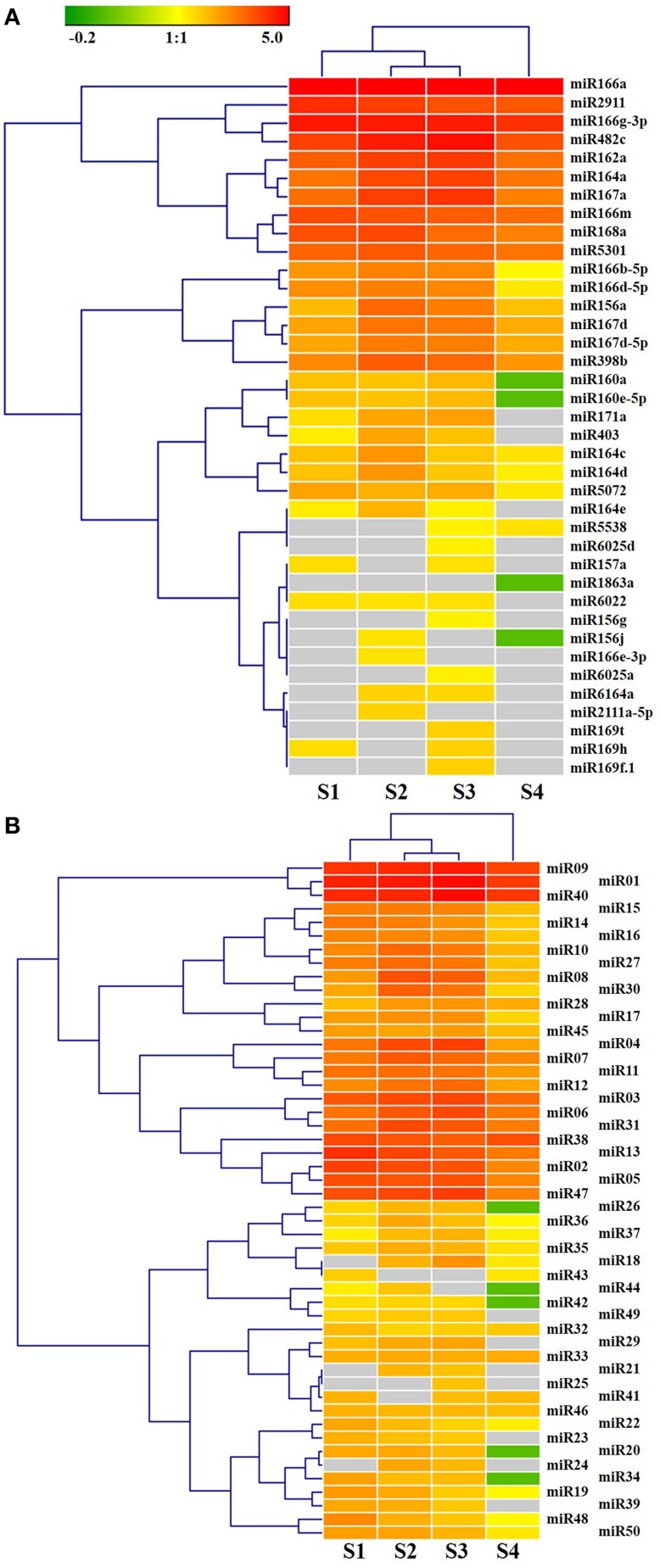
**Hierarchical clustering analysis of known miRNAs (A) and novel miRNAs (B) differentially expressed in ripening fruit of *Lycium barbarum* L**. The expression profiles were analyzed by Genesis software with hierarchical clustering method based on miRNAs TPM Log10-transformed. Gray box indicate miRNA transcript were not detected in the corresponding samples.

As shown in Figure 3, miR166e-3p and miR2111a-5p were stage-specifically expressed in S2 and miR1863a was specific to S4. Six S3-specific miRNAs were identified, comprising miR169f.1/t, miR6025a/d, miR156g, and miR25. Eight miRNAs were common to S1, S2 and S3 but not S4; these were miR23, miR29, miR39, miR49, miR171a, miR6022, miR164e, and LbamiR403. In addition, three miRNAs (miR6164a, miR21, and miR24) were only expressed in S2 and S3, while miR5538 was specific to the late stages S3 and S4. The remaining 60 miRNAs were differentially expressed in all stages (Figure 3C). As shown in Figure S6, miRNAs were expressed abundantly in S1 and S2 and decreased in S3 and S4 during fruit ripening. Among the known miRNAs, miR166a, miR2911, miR166g-3p, miR482c, miR162a, miR164a, miR167a, miR166m, miR168a, and miR5301 were consistently more highly expressed during fruit ripening than miR5538, miR6025d, miR157a, miR1863a, miR6022, miR156g, miR156j, miR166e-3p, miR6025a, miR6164a, miR2111a-5p, miR169t, miR169h, and miR169f.1 (Figure 4A). The latter miRNAs were the stage-specific miRNAs discussed above, and were expressed at a low level.

As shown in Figure 4A, different members of the same miRNA family showed complete divergence in expression pattern and expression level, for instance miR166a vs. miR166e-3p as well as miR164a vs. miR164c/d. miR164 members were predicted here to regulate overlapping as well as distinct target genes (Figure S7), suggesting that some miRNA family members might be functionally divergent. In Arabidopsis, miR164 family members miR164a-c show both over-lapping and diverse functions (Mallory et al., 2004; Baker et al., 2005; Guo et al., 2005; Nikovics et al., 2006; Sieber et al., 2007). Several novel miRNAs, for instance miR01, miR40, miR09, miR13, miR38, and miR47, were expressed as highly as that of known miRNAs (Figure 4B). The remaining novel miRNAs were expressed at a low level. Noticeably, 14 novel miRNAs were also detected in *L. chinense* (Table 2).

### Prediction of putative target genes for known and novel miRNAs

A total of 441 unigenes were predicted to be targets of miRNAs using PsRobot software (Table S5). A number of miRNAs had multiple putative target genes, ranging from 1 to 116 (for LbmiR6164). Inversely, several putative target genes were targeted by multiple miRNAs with up to five miRNAs predicted to target Poly (A) RNA polymerase cid14 (Figure S8). Noticeably, only 3 out of the putative 50 novel miRNAs (miR02, miR24, and miR28) were successfully predicted to target *L. barbarum* unigenes (Table S5). miR02 and miR24 were predicted to target a late blight resistance gene and a histone H2B.1-like gene, respectively, while miR28 targeted a gene with unknown function (Table S5). As shown in Figure S9 and Table S5, a relatively high proportion of target genes were annotated as TFs. For instance, miR160, miR156, and miR164 putatively target auxin response factors (ARF), SPL, and NAC TFs, respectively. Similarly, miR169 was predicted to target NF-YA, while miR171 and miR157 may target GRAS and MADS-box TFs, respectively. Aside from TFs, targets of miRNAs (e.g., miR6025, miR169, miR156, and miR482) were involved in resistance to disease or virus attack, suggesting that miRNAs are involved in regulation of the *L. barbarum* defense response. In addition, miR168, miR403, and miR6164 were predicted to target genes encoding proteins involved in miRNA biogenesis including AGO1, AGO2, and RNA-directed DNA polymerase. This suggests that small RNA-mediated silencing may be regulated in a feedback manner. Furthermore, pyruvate kinase (*PK*), fructokinase (*FK*), and beta-fructofuranosidase (β*-FFase*) involved in glycolysis and sucrose metabolism were also predicted to be regulated by miRNAs (miR157, miR156, and miR164, respectively). Two miRNAs were predicted to be involved in carotenoid biosynthesis, for instance miR156 was predicted to target 1-deoxy-D-xylulose-5-phosphate synthase (*DXS*), with miR6022 putatively regulating DNA damage-binding protein 1 (*DDB1*). Five unigenes predicted to be targeted by miR156/157 and one unigene by miR2111 were involved in ubiquitin-mediated proteolysis (Table S5).

**Figure 5:**
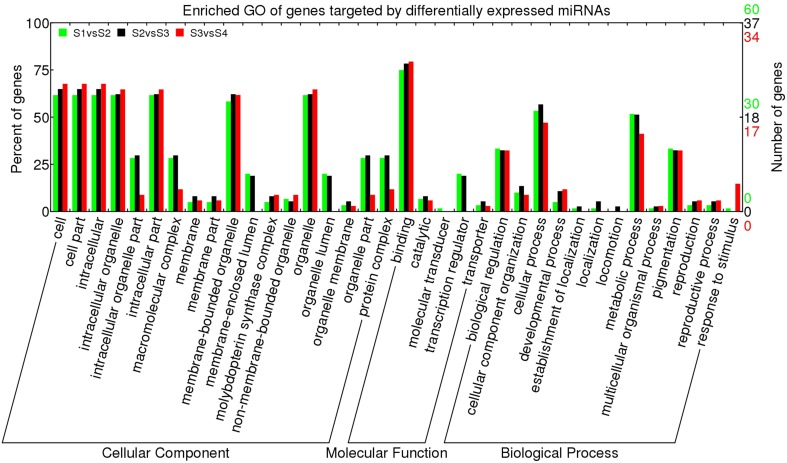
**Enriched GO term of genes targeted by differentially expressed miRNAs in *Lycium barbarum* ripening fruits**. The differentially expressed miRNAs were defined as following the criteria of *q* < 0.01 and |log2(foldchange)| > 1 based on the number of miRNA reads in small RNA libraries. Enriched GO was plotted using WEGO (http://wego.genomics.org.cn/cgi-bin/wego/index.pl).

To gain a global overview of the regulatory functions of miRNAs, the GO terms of all targets were analyzed. Among the 441 target genes, 194 target genes had GO terms. As shown in Figure S9, the major “cellular components” predicted for these GO-defined target genes were “cell part,” “organelle,” “intracellular,” “intracellular organelle,” “intracellular part,” and “membrane-bounded organelle.” The main molecular functions of target genes were classified as “binding,” “catalytic,” “transcription regulator,” and “transporter,” while the key biological processes were “cellular process,” “metabolic process,” “biological regulation,” and “pigmentation.” In order to better understand the regulatory role of miRNA expressed during *L. barbarum* fruit ripening, the enriched GO terms of target genes of differentially expressed miRNAs were analyzed in S1 vs. S2 (S1 vs. S2), S2 vs. S3, or S3 vs. S4 (Figure 5). As shown in Figure 5, the distribution of enriched GO terms of targets of differentially regulated miRNAs was similar for all adjacent stages, with the major cellular components of “cell part,” “intracellular,” “intracellular organelle,” “organelle,” “intracellular part,” and “membrane-bounded organelle.” The main molecular function classification was “binding” and the chief biological processes were “cellular process,” “metabolic process,” “pigmentation” as well as “biological regulation.” Noticeable exceptions were “intracellular organelle part,” “macromolecular complex,” and “transcription regulator” significantly decreased in S3 vs. S4 when compared to S1 vs. S2 or S2 vs. S3.

To universally summarize the orchestrating roles of miRNAs in *L. barbarum* fruit ripening, enriched KEGG analysis of target genes of differentially expressed miRNAs was performed. There were 4, 4, and 5 pathways over-represented respectively during the transition from S1 to S2, S2 to S3, and S3 to S4 (Table 3). From S1 to S2, nucleotide excision repair and ubiquitin mediated proteolysis were most over-represented, while ABC transporters and sulfur metabolism pathways were most active in the change from S2 to S3. From S3 to S4, ABC transporters, spliceosome, and GABAergic synapse pathways were most altered. Furthermore, fatty acid biosynthesis (*P* = 0.055) and terpenoid backbone biosynthesis (*P* = 0.061) were over-represented, although not statistically.

**Table 3:** **Enriched KEGG pathways revealed by unigenes targeted by differentially expressed miRNAs**.

	**KO ID**	**Term**	***P*-Value**
S1 vs. S2	ko04612	Antigen processing and presentation	0.0000
	ko03420	Nucleotide excision repair	0.0090
	ko04120	Ubiquitin mediated proteolysis	0.0093
	ko00590	Arachidonic acid metabolism	0.0490
S2 vs. S3	ko04612	Antigen processing and presentation	0.0000
	ko02010	ABC transporters	0.0335
	ko00760	Nicotinate and nicotinamide metabolism	0.0404
	ko00920	Sulfur metabolism	0.0499
S3 vs. S4	ko04962	Vasopressin-regulated water reabsorption	0.0139
	ko02010	ABC transporters	0.0208
	ko03040	Spliceosome	0.0223
	ko04727	GABAergic synapse	0.0335
	ko00460	Cyanoamino acid metabolism	0.0361
	ko00061	Fatty acid biosynthesis	0.0553
	ko00900	Terpenoid backbone biosynthesis	0.0611

### Validation of miRNA and target gene expression

**Figure 6:**
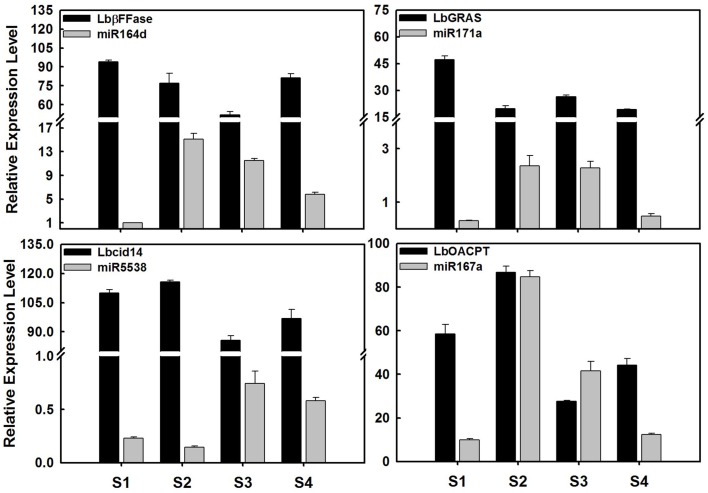
**Expression profile of miRNAs and their target genes in ripening fruits of *Lycium barbarum* L**. The total RNAs were isolated from fruits at four developmental stages referring to S1 stage (green fruit, 3 days before color break), S2 stage (the color-break stage), S3 stage (light-color, 3 days after color break), and S4 stages (ripe fruit, 6 days after color break). These results were presented as mean ± SD of three biological replicates. The *Lb*β*FFase* is predicted to be the target gene of miR164d, as well as *LbGRAS* for miR171a, *Lbcid14* for miR5538, and *LbOACPT* for miR167a. The expression profile of miR164d, miR171a, miR167a, and miR5538 tested by qRT-PCR was identical to that of small RNA sequencing.

**Figure 7:**
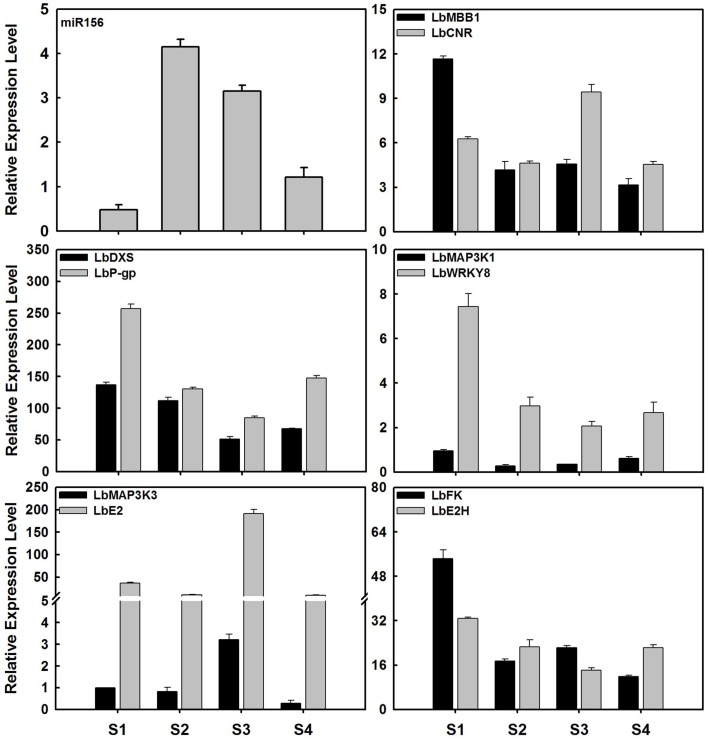
**Expression profile of miR156 and its target genes in ripening fruits of *Lycium barbarum* L**. The total RNAs were isolated from fruits at four developmental stages referring to S1 stage (green fruit, 3 days before color break), S2 stage (the color-break stage), S3 stage (light-color, 3 days after color break), and S4 stages (ripe fruit, 6 days after color break). These results were presented as mean ± SD of three biological replicates. The miR156 expression level presented here represented overall level of all miR156 members. The expression profile of miR156 tested by qRT-PCR was identical to that of sequencing of small RNA libraries.

To validate the expression profiles of miRNAs in ripening fruits of *L. barbarum*, 13 significant miRNAs were investigated using stem-loop qRT-PCR (Figures 4, 6, 7 and Figure S10). Among these, the expression profiles of miR164d, miR171a, miR167a, miR403, miR5538, miR09, and miR40 were consistent with the results of small RNA sequencing (Figures 4, 6, 7). As shown in Figures 6, 7, miR164d, miR171a, miR167a, and miR156 transcripts were expressed highly in S2 and decreased subsequently. In contrast, miR5538 transcripts decreased from S1 to S2, then increased to the highest level at S3 followed by decrease at S4.

In this study, it was difficult to distinguish the expression level of different members of miR156 when using qRT-PCR. Both stem-loop qRT-PCR and small RNA sequencing showed the identical expression pattern of miR156 in ripening fruits. The remaining five miRNAs tested, including miR01, miR02, miR13, miR28, and miR168, showed inconsistent results between stem-loop qRT-PCR and small RNA sequencing (Figure S10 and Tables 1, 2). For instance, small RNA sequencing suggested that miR168a transcripts decreased during *L. barbarum* fruit ripening, while the opposite trend was shown by qRT-PCR. This might be partially attributed to only two biological replicates being respectively used for stem-loop qRT-PCR and small RNA sequencing.

In order to validate the candidate targets of miRNAs, the expression profiles of protein-coding genes targeted by miRNAs was also investigated in ripening fruits. *Lb*β*FFase* (targeted by miR164d), *LbGRAS* (miR171a), *Lbcid14* (miR5538), *LbOACPT* (miR167a) and ten targets of LbamiR156 members were analyzed by qRT-PCR (Figures 6, 7). As shown in Figure 6, the expression profiles of miR164d, miR171a, miR5538, and miR167a showed opposite expression trends to their target genes, as expected for miRNA targets. The same was shown for putative targets of LbmiR156, including *LbMBB1, LbDXS, LbP-gp, LbMAP3K1, LbWRKY8, LbFK*, and *LbE2H*. Our results suggest that these candidate genes might be *bona fide* target genes of the miRNAs tested here. However, three of the putative LbmiR156 targets; *LbCNR, LbMAP3K3*, and *LbE2* did not show predicted expression patterns for miR156 targets (Figure 7), suggesting they may not be cleaved by this miRNA. Because qRT-PCR primers used in this study flanked the miRNA target site, it is possible that the activity of these genes is determined by translational repression and/or by multiple miRNAs.

## Discussion

Small RNAs including miRNAs are key regulators of biological processes, including biotic and abiotic stress tolerance, plant growth and development, metabolic pathways, and morphogenesis. It is well documented that miRNAs orchestrate fruit development in multiple crop plants including tomato (Chen et al., 2015), pear (Wu et al., 2014), orange (Xu et al., 2010), and persimmon (Luo et al., 2015). Although miRNAs have been identified in *L. chinense* fruits and shoot tips using small RNA sequencing, miRNAs in *L. barbarum* fruits have not yet been characterized. Our previous study documents that *L. barbarum* fruits increase in carotenoid content during fruit ripening (Liu et al., 2014b), suggesting that fruit ripening involves modulation of fruit pigmentation in *L. barbarum*. Therefore, identification and characterization of miRNAs in *L. barbarum* fruits may provide valuable information for better understanding the biological process of fruit ripening, including fruit development, pigmentation and quality.

In this study, four fruit samples taken sequentially during development were analyzed, namely green fruit, color-break fruit, light-yellow fruit, and red fruit. This resulted in characterization of 50 novel and 38 known miRNAs across 22 families. Of 441 predicted target unigenes, 194 were successfully annotated (Table S5). The number of predicted target genes of each miRNA ranged from 1 to 169, seen for miR6164a. In pear fruit, the largest number of target genes described was 226 for miR396b, followed by 149 for miR5564 and 137 for miR4993 (Wu et al., 2014). The unexpectedly large numbers of target genes might be partially attributed to mature miRNA sequences showing high identity with repetitive motifs (Bonnet et al., 2004).

As shown in Figure 3, several stage-specific miRNAs were identified. For instance, miR166e-3p and miR2111a-5p, and miR1863a transcripts were restricted to S2 and S4, respectively. Six miRNAs, including miR169f, miR169t, miR6025a, miR6025d, miR156g, and miR25, were stage-specifically expressed in S3. As shown in Figure 6, *LbGRAS* is predicted to be targeted by miR171a, which is expressed increasingly from S1 to S3 while not in stage S4, suggesting a possible role for both genes in the ripening process. These results also suggest that these miRNAs might govern stage-specific developmental transitions of ripening fruit.

Previous studies reveal that miR164 targets a set of NAC TFs (Mallory et al., 2004; Guo et al., 2005; Nikovics et al., 2006). Here, miR6164a was predicted to target a *LbNAC* TF homologous to *SlNAC4*. In tomato, *SlNAC4* RNAi-knockout plants have delayed fruit ripening (Zhu et al., 2014), suggesting that *SlNAC4* is a positive regulator of ripening. Furthermore, a reduction of 30% total carotenoid in *SlNAC4* RNAi lines were detected in both pericarp and placenta when compared to control lines (Zhu et al., 2014), suggesting that *SlNAC4* functions as a positive regulator of carotenoid accumulation. Consequently, miR6164a might be a novel miRNA involved in *L. barbarum* fruit ripening and pigmentation. In this study, three unigenes encoding NF-YA TFs were predicted to be targeted by the S3-specific miRNA miR169 (Table S5). In petunia and Antirrhinum, miR169 depresses C class MADS box genes involved in carpel development by inhibiting the expression of NF-YA TFs (Cartolano et al., 2007). This may suggest that miR169 is involved in fruit development.

The miRNA modulation of fruit ripening has gained much attention in tomato, a model species in fruit development study. Recently, fruit ripening in tomato was correlated with modulation of *SlCNR* expression by miR157 in a dose-dependent manner, through both mRNA cleavage and translational repression (Chen et al., 2015). Furthermore, miR156 was shown to control the tomato fruit softening process and orchestrate the initial steps of fruit determinacy and development (Ferreira e Silva et al., 2014; Chen et al., 2015). As shown in Table S5, *LbCNR* is the putative target of both miR157 and miR156. Here, however, *LbCNR* expression pattern did not inversely correlate to miR156 (Figure 7). However, as *LbCNR* expression was evaluated using primers flanking the miRNA target site, this might be partially attributed to a regulatory tradeoff of mRNA cleavage and/or translational repression conducted by miR157 and/or miR156. As shown in Figure 7, miR156 targeted ten candidate genes belonging to different gene families. These include the aforementioned TF *LbCNR*, an additional TF-family member *LbWRKY8*, the signal transduction family genes *LbMAP3Ks* and *Lb2Es*, chloroplastic *LbMBB1* involved in *psbB* mRNA maturation, as well as the plasma membrane proteins *LbDXS* and *LbP-gp*. Specifically, *LbWRKY8*, showed an inverse expression relationship to miR156 during ripening, suggesting that it might be a negative regulator of ripening. In tomato, *DXS* coordinating with *PSY1* controls carotenoid synthesis during fruit ripening (Lois et al., 2000). Consequently, it was reasonable to postulate that these miR156 target genes might be involved in the same biological process of *L. barbarum* fruit ripening although these target genes belonged to different gene families. A similar case is documented in tomato, where miR-W^*^ targets two membrane bound proteins (ATPase and glutamate permease) belonging to two different gene families but both involvement in ATP-dependent glutamate transport (Mohorianu et al., 2011).

**Figure 8:**
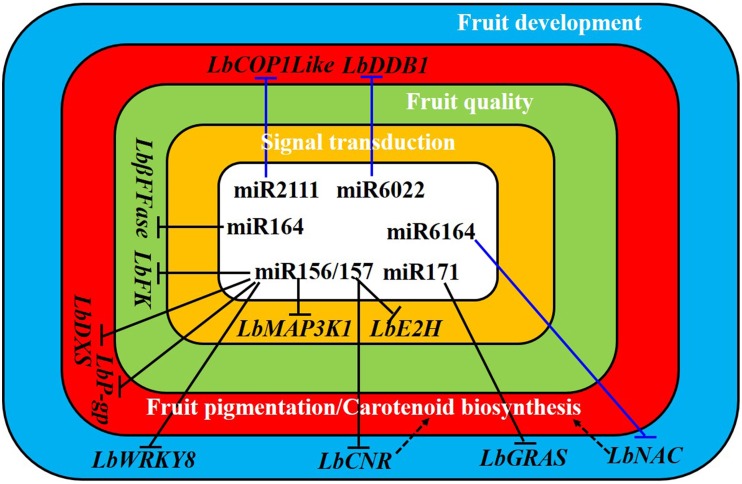
**A hypothetical model underlying of miRNA modulation of fruit ripening in *Lycium barbarum* L**. The black and blue “T” signs denote the interactions among miRNA-target gene investigated and predicted in this study, respectively. Dashed arrows indicate the potential function of *LbCNR* and *LbNAC* homologous to *SlNAC4* in controlling carotenoid biosynthesis. In tomato, both *SlCNR* (Fraser et al., 2001) and *SlNAC4* (Zhu et al., 2014) are evidenced to be positive regulators in carotenoid biosynthesis while *SlCOP1like* (Liu et al., 2004) and *SlDDB1* (Wang et al., 2008) are negative regulators in fruit pigmentation. *LbFK, L. barbarum* fructokinase; *LbDXS, L. barbarum* 1-deoxy-D-xylulose-5-phosphate synthase; *Lb*β*FFase, L. barbarum* beta-fructofuranosidase; *L. barbarum* P-glycoprotein; *LbMAP3K1, L. barbarum* mitogen-activated protein kinase kinase kinase 1; *LbE2H, L. barbarum* ubiquitin-conjugating enzyme E2H.

The *L. barbarum* homologs for two additional genes involved in carotenoid synthesis and fruit pigmentation during ripening, *COP1like* and *DDB1*, were also targetted by ripening stage-specific miRNAs in this study; miR2111a-5p (S2-specific) and miR6022 (specific in stages from S1 to S3), respectively (Table S5). *DDB1* RNAi knockouts in tomato show enhanced numbers of plastids and pigment accumulation (Wang et al., 2008). Furthermore, repression of *SlCOP1like* results in increased carotenoid content in transgenic tomato fruits, suggesting that *SlCOP1like* functions as a negative regulator of fruit pigmentation (Liu et al., 2004). In tomato, the *cnr* mutation results in fruit-specific low levels of total carotenoid (Fraser et al., 2001). In this study, miR156/157 was also predicted to target *LbCNR*, suggesting that *LbCNR* may affect carotenoid biosynthesis in *L. barbarum* fruit. As such, the *Lycium* homologs to genes and their regulatory miRNAs in tomato might fulfill similar functions to determine fruit pigmentation. The glycolysis and sucrose metabolism-related genes *FK, PK*, and β*FFase* were predicted targets of miR156, miR157, and miR164, suggesting that these miRNAs may be involved in modulating fruit quality. Overall, miRNAs are master modulators, orchestrating certain metabolic, developmental or physiological processes by regulating both secondary regulators (i.e., TFs) and other related genes (i.e., biosynthetic genes) involved in the same biological event, for instance fruit ripening (Figure 8).

In tomato, both miR172 and miR390 are lowly expressed in ripening fruits while miR156, miR164, and miR396 are upregulated (Mohorianu et al., 2011). miR159, miR165/166, and miR162 are predominant in early fruit development before color break (Mohorianu et al., 2011). In *L. barbarum* ripening fruit, miR162, miR164, miR167 and miR482 were upregulated and miR166 and miR2911 were downregulated in stages from green color (S1) through color break (S2) to maturing-color (S3). As shown in Figure 7 and Table 1, miR156 transcripts were sharply enhanced at S2 compared to S1 and decreased from S2 to S4. Therefore, miR165/166 and miR164 show conserved expression profiles in both tomato and *L. barbarum* ripening fruits. On the other hand, miR162 and the ripening-related miR156 appear to show divergent expression profiles between these two Solanaceous fruits. These results suggest that the miRNA-mediated control of *L. barbarum* fruit development might be different to a certain extent to that of tomato on the basis of distinct expression profiles.

A large number of novel miRNAs have also been identified in other fruits including persimmon (Luo et al., 2015), *L. chinense* (Khaldun et al., 2015), and pear (Wu et al., 2014). In this study, 39 novel miRNAs, whose miRNA^*^ counterparts were also detected in fruit samples, were successfully identified. Of the 30 novel *L. chinense* miRNAs previously identified, 14 were detected here in *L. barbarum*. These results indicate that 36 and 16 species-specific miRNAs exist in *L. barbarum* and *L. chinense*, respectively. Parsimony analyses of both nuclear *waxy* data and chloroplastic *trnT-trnF* data indicate that *L. barbarum* is the closest relative species of *L. chinense* in genus *Lycium* (Levin and Miller, 2005). This result suggests that miRNA evolution in genus *Lycium* is rapid, which is consistent with Arabidopsis (Fahlgren et al., 2010). At the genome-wide level, at least 13% of *MIR* genes are species-specific in two related Arabidopsis species (Fahlgren et al., 2010). Thus, it appears that *MIR* genes may evolve rapidly so that certain miRNA(s) are species-specific (Chen, 2009; Cuperus et al., 2011).

In contrast to conserved miRNAs, novel miRNAs with low abundance may appear to lack targets based on current criteria, and may also be instantaneous and non-functional (Fahlgren et al., 2010; Bologna and Voinnet, 2014). In Arabidopsis, evolutionarily young miRNAs are expressed at low levels and few have known targets (Fahlgren et al., 2010). In this study, only three novel miRNAs (miR02, miR24, and miR28) were successfully predicted to target protein-coding genes, suggesting that most novel miRNAs might be non-functional or have as yet undetermined roles in responding to specific physiological or developmental events. Our qRT-PCR assay was not able to detect five out of six lowly-expressed novel miRNAs (miR03, miR05, miR07, miR16, miR17, and miR24) (Data not shown). Additionally, miR16 (5′-AAGCCUUCCGCAAUCCAUAAC-3′) and miR05 (5′-AAUCCUUCUGCAAUCCAUAAC-3′) were derived from the same transcript but predicted to be divergent due to RNA editing. The regulatory power of miRNAs over their target genes can fluctuate according to both difference in precursor processing efficiency and subtle changes in the mature miRNA sequence itself (Bologna and Voinnet, 2014). Taken together, the species-specificity and low level of expression of predicted novel miRNAs, combined with RNA editing and lack of predicted targets, indicated that these novel miRNAs expressed in ripening fruit may have evolved neutrally in the *Lycium* genus and play an undetermined role in fruit ripening.

In conclusion, using Illumina HiSeq™ 2000 sequencing, 50 novel miRNAs and 38 known miRNAs were identified in *L. barbarum* fruits. Of the novel miRNAs, 36 were not detected in the closely related *L. chinense*, suggesting that young miRNAs may evolve rapidly in *Lycium* genus. A number of stage-specific LbamiRNAs were respectively identified in S1, S2, S3, and S4, suggesting stage-specific regulatory functions. These miRNAs included S2-specific miR166e-3p and miR2111a-5p, S4-specific miR1863a and six S3-specific miRNAs (miR169f, miR169t, miR6025a, miR6025d, miR156g, and miR25). Several candidate miRNAs and their target genes were predicted and/or identified that have known function in determining fruit quality fruit development, and fruit pigmentation. For instance, putative carotenoid-related gene *LbCOP1like* and *LbDDB1* were predicted to be the target of S2-specific miR2111 and miR6022 only expression in S1–S3 stages, respectively. Furthermore, candidate genes involved in fruit development and fruit pigmentation, *LbNAC* and *LbCNR*, were predicted to be the target of miR6164 and miR156/157, respectively. Another two fruit development regulators (*LbWRKY8* and *LbGRAS*) and their regulatory miRNAs (miR156 and miR171) were validated by qRT-PCR. Thus, these interactions may play master regulatory roles in modulating *L. barbarum* fruit ripening (Figure 8). In all, the identification of miRNAs and their putative target interactions during ripening provides a solid foundation for uncovering the miRNA-mediated mechanism of fruit ripening.

### Conflict of interest statement

The authors declare that the research was conducted in the absence of any commercial or financial relationships that could be construed as a potential conflict of interest.

## Supplementary material

Table S1 **List of primers used for stem-loop reverse transcription and qRT-PCR**. URP indicate the universe reverse primer used for miRNA qRT-PCR.

Table S2 **Sequence summary of high quality small RNA filtered**. “a” and “b” indicate that raw and unique reads of small RNA mapped to *Lycium barbarum* transcriptome, respectively.

Table S3 **Classification and annotation of small RNA mapped to *Lycium barbarum* L. transcriptome**. TAS, Trans-acting siRNA.

Table S4 **Expression profile of miRNAs^*^ in ripening fruits**. The expression profile of miRNA/miRNA^*^ were presented in the number of raw reads. “/” indicate that no read was detected in the four libraries. P1, P2, and P3 indicated these LbamRNAs derived from genes encoding glutathione S-transferase T1-like, unnamed protein, and simiate protein, respectively.

Table S5 **Annotation of miRNA target genes in *Lycium barbarum* L**.

Figure S1 **Repeat classification of total (A) and unique (B) sRNA reads**.

Figure S2 **Summary of total (A) or unique (B) small RNA shared in or specific to sample(s)**.

Figure S3 **Predicted secondary structures of novel miRNAs**.

Figure S4 **First nucleotide biased in known miRNA (A) and novel miRNA (B)**.

Figure S5 **Nucleotide biased at each position of known miRNA (A) and novel miRNA (B)**.

Figure S6 **Density distribution of miRNA transcripts in ripening fruits of *Lycium barbarum* L**. The expression level of miRNAs were Log10-transformed.

Figure S7 **miR164 members diversely target different unigenes**.

Figure S8 **Summary of the number of unigenes targeted by one miRNAs (A) and the number of miRNA jointly targeting one unigenes (B)**.

Figure S9 **GO term of unigenes targeted by all miRNAs detected in ripening fruits**. GO terms were plotted using WEGO (http://wego.genomics.org.cn/cgi-bin/wego/index.pl).

Figure S10 **Expression profile of miRNAs in ripening fruits of *Lycium barbarum* L**. The total RNAs were isolated from fruits at four developmental stages referring to S1 stage (green fruit, 3 days before color break), S2 stage (the color-break stage), S3 stage (light-color, 3 days after color break), and S4 stages (ripe fruit, 6 days after color break). These results were presented as mean ± SD of three biological replicates. The expression profile of miR09, miR40, and miR403 tested by qPCR was identical to that of sequencing of small RNA libraries. While the results of expression profile of residual *Lycium barbarum* miRNAs were distinctly revealed by qRT-PCR and small RNA library sequencing.

## References

[B1] Amagase H. Farnsworth N. R. (2011). A review of botanical characteristics, phytochemistry, clinical relevance in efficacy and safety of *Lycium barbarum* fruit (Goji). Food Res. Int. 44, 1702–1717. 10.1016/j.foodres.2011.03.027

[B2] Baker C. C. Sieber P. Wellmer F. Meyerowitz E. M. (2005). The early extra petals1 mutant uncovers a role for microRNA miR164c in regulating petal number in *Arabidopsis*. Curr. Biol. 15, 303–315. 10.1016/j.cub.2005.02.017 15723790

[B3] Bologna N. G. Voinnet O. (2014). The diversity, biogenesis, and activities of endogenous silencing small RNAs in *Arabidopsis*. Annu. Rev. Plant Biol. 65, 473–503. 10.1146/annurev-arplant-050213-035728 24579988

[B4] Bonnet E. Wuyts J. Rouzé P. Van De Peer Y. (2004). Detection of 91 potential conserved plant microRNAs in *Arabidopsis thaliana* and *Oryza sativa* identifies important target genes. Proc. Natl. Acad. Sci. U.S.A. 101, 11511–11516. 10.1073/pnas.0404025101 15272084 PMC509231

[B5] Cartolano M. Castillo R. Efremova N. Kuckenberg M. Zethof J. Gerats T.. (2007). A conserved microRNA module exerts homeotic control over *Petunia hybrida* and *Antirrhinum majus* floral organ identity. Nat. Genet. 39, 901–905. 10.1038/ng2056 17589508

[B6] Chen W. Kong J. Lai T. Manning K. Wu C. Wang Y.. (2015). Tuning LeSPL-CNR expression by SlymiR157 affects tomato fruit ripening. Sci. Rep. 5:7852. 10.1038/srep07852 25597857 PMC4297963

[B7] Chen X. (2009). Small RNAs and their roles in plant development. Annu. Rev. Cell Dev. Biol. 25, 21–44. 10.1146/annurev.cellbio.042308.113417 19575669 PMC5135726

[B8] Chuck G. Cigan A. M. Saeteurn K. Hake S. (2007). The heterochronic maize mutant *Corngrass1* results from overexpression of a tandem microRNA. Nat. Genet. 39, 544–549. 10.1038/ng2001 17369828

[B9] Chung M. Y. Vrebalov J. Alba R. Lee J. McQuinn R. Chung J. D.. (2010). A tomato (*Solanum lycopersicum*) APETALA2/ERF gene, *SlAP2a*, is a negative regulator of fruit ripening. Plant J. 64, 936–947. 10.1111/j.1365-313X.2010.04384.x 21143675

[B10] Cuperus J. T. Fahlgren N. Carrington J. C. (2011). Evolution and functional diversification of miRNA genes. Plant Cell 23, 431–442. 10.1105/tpc.110.082784 21317375 PMC3077775

[B11] Eriksson E. M. Bovy A. Manning K. Harrison L. Andrews J. De Silva J.. (2004). Effect of the colorless non-ripening mutation on cell wall biochemistry and gene expression during tomato fruit development and ripening. Plant Physiol. 136, 4184–4197. 10.1104/pp.104.045765 15563627 PMC535848

[B12] Fahlgren N. Jogdeo S. Kasschau K. D. Sullivan C. M. Chapman E. J. Laubinger S.. (2010). MicroRNA gene evolution in *Arabidopsis lyrata* and *Arabidopsis thaliana*. Plant Cell 22, 1074–1089. 10.1105/tpc.110.073999 20407027 PMC2879733

[B13] Ferreira e Silva G. F. Silva E. M. Azevedo Mda S. Guivin M. A. Ramiro D. A. Figueiredo C. R.. (2014). microRNA156-targeted SPL/SBP box transcription factors regulate tomato ovary and fruit development. Plant J. 78, 604–618. 10.1111/tpj.12493 24580734

[B14] Fraser P. D. Bramley P. Seymour G. B. (2001). Effect of the *Cnr* mutation on carotenoid formation during tomato fruit ripening. Phytochemistry 58, 75–79. 10.1016/S0031-9422(01)00175-3 11524116

[B15] Friedländer M. R. Mackowiak S. D. Li N. Chen W. Rajewsky N. (2012). miRDeep2 accurately identifies known and hundreds of novel microRNA genes in seven animal clades. Nucleic Acids Res. 40, 37–52. 10.1093/nar/gkr688 21911355 PMC3245920

[B16] Fujisawa M. Nakano T. Shima Y. Ito Y. (2013). A large-scale identification of direct targets of the tomato MADS box transcription factor RIPENING INHIBITOR reveals the regulation of fruit ripening. Plant Cell 25, 371–386. 10.1105/tpc.112.108118 23386264 PMC3608766

[B17] Fujisawa M. Shima Y. Higuchi N. Nakano T. Koyama Y. Kasumi T.. (2012). Direct targets of the tomato-ripening regulator RIN identified by transcriptome and chromatin immunoprecipitation analyses. Planta 235, 1107–1122. 10.1007/s00425-011-1561-2 22160566

[B18] Gao C. Ju Z. Cao D. Zhai B. Qin G. Zhu H.. (2015). MicroRNA profiling analysis throughout tomato fruit development and ripening reveals potential regulatory role of RIN on microRNAs accumulation. Plant Biotechnol. J. 13, 370–382. 10.1111/pbi.12297 25516062

[B19] Guo H. Xie Q. Fei J. Chua N. (2005). MicroRNA directs mRNA cleavage of the transcription factor NAC1 to downregulate auxin signals for Arabidopsis lateral root development. Plant Cell 17, 1376–1386. 10.1105/tpc.105.030841 15829603 PMC1091761

[B20] Kanehisa M. Araki M. Goto S. Hattori M. Hirakawa M. Itoh M.. (2008). KEGG for linking genomes to life and the environment. Nucleic Acids Res. 36, D480–D484. 10.1093/nar/gkm882 18077471 PMC2238879

[B21] Karlova R. van Haarst J. C. Maliepaard C. van de Geest H. Bovy A. G. Lammers M.. (2013). Identification of microRNA targets in tomato fruit development using high-throughput sequencing and degradome analysis. J. Exp. Bot. 64, 1863–1878. 10.1093/jxb/ert049 23487304 PMC3638818

[B22] Khaldun A. B. M. Huang W. Liao S. Lv H. Wang Y. (2015). Identification of microRNAs and target genes in the fruit and shoot tip of *Lycium chinense*: a traditional chinese medicinal plant. PLoS ONE 10:e0116334. 10.1371/journal.pone.0116334 25587984 PMC4294688

[B23] Khvorova A. Reynolds A. Jayasena S. D. (2003). Functional siRNAs and miRNAs exhibit strand bias. Cell 115, 209–216. 10.1016/S0092-8674(03)00801-8 14567918

[B24] Levin R. A. Miller J. S. (2005). Relationships within tribe Lycieae (Solanaceae): paraphyly of *Lycium* and multiple origins of gender dimorphism. Am. J. Bot. 92, 2044–2053. 10.3732/ajb.92.12.2044 21646122

[B25] Liu Y. Roof S. Ye Z. Barry C. van Tuinen A. Vrebalov J.. (2004). Manipulation of light signal transduction as a means of modifying fruit nutritional quality in tomato. Proc. Natl. Acad. Sci. U.S.A. 101, 9897–9902. 10.1073/pnas.0400935101 15178762 PMC470770

[B26] Liu Y. Wang L. Chen D. Wu X. Huang D. Chen L.. (2014a). Genome-wide comparison of microRNAs and their targeted transcripts among leaf, flower and fruit of sweet orange. BMC Genomics 15:695. 10.1186/1471-2164-15-695 25142253 PMC4158063

[B27] Liu Y. Zeng S. Sun W. Wu M. Hu W. Shen X.. (2014b). Comparative analysis of carotenoid accumulation in two goji (*Lycium barbarum* L. and *L. ruthenicum* Murr.) fruits. BMC Plant Biol. 14:269. 10.1186/s12870-014-0269-4 25511605 PMC4276078

[B28] Livak K. J. Schmittgen T. D. (2001). Analysis of relative gene expression data using real-time quantitative PCR and the 2^−Δ*ΔCT*^ method. Methods 25, 402–408. 10.1006/meth.2001.1262 11846609

[B29] Lois L. M. Rodríguez-Concepción M. Gallego F. Campos N. Boronat A. (2000). Carotenoid biosynthesis during tomato fruit development: regulatory role of 1-deoxy-D-xylulose 5-phosphate synthase. Plant J. 22, 503–513. 10.1046/j.1365-313x.2000.00764.x 10886770

[B30] Luo Y. Zhang X. Luo Z. Zhang Q. Liu J. (2015). Identification and characterization of microRNAs from Chinese pollination constant non-astringent persimmon using high-throughput sequencing. BMC Plant Biol. 15:11. 10.1186/s12870-014-0400-6 25604351 PMC4308916

[B31] Mallory A. C. Dugas D. V. Bartel D. P. Bartel B. (2004). Microrna regulation of NAC-domain targets is required for proper formation and separation of adjacent embryonic, vegetative, and floral organs. Curr. Biol. 14, 1035–1046. 10.1016/j.cub.2004.06.022 15202996

[B32] Mao X. Cai T. Olyarchuk J. G. Wei L. (2005). Automated genome annotation and pathway identification using the KEGG Orthology (KO) as a controlled vocabulary. Bioinformatics 21, 3787–3793. 10.1093/bioinformatics/bti430 15817693

[B33] Martinez J. Tuschl T. (2004). RISC is a 5′ phosphomonoester-producing RNA endonuclease. Genes Dev. 18, 975–980. 10.1101/gad.1187904 15105377 PMC406288

[B34] Mi S. Cai T. Hu Y. Chen Y. Hodges E. Ni F.. (2008). Sorting of small RNAs into *Arabidopsis* argonaute complexes is directed by the 5′ terminal nucleotide. Cell 133, 116–127. 10.1016/j.cell.2008.02.034 18342361 PMC2981139

[B35] Mohorianu I. Schwach F. Jing R. Lopez-Gomollon S. Moxon S. Szittya G.. (2011). Profiling of short RNAs during fleshy fruit development reveals stage-specific sRNAome expression patterns. Plant J. 67, 232–246. 10.1111/j.1365-313X.2011.04586.x 21443685

[B36] Nikovics K. Blein T. Peaucelle A. Ishida T. Morin H. Aida M.. (2006). The balance between the miR164a and CUC2 genes controls leaf margin serration in Arabidopsis. Plant Cell 18, 2929–2945. 10.1105/tpc.106.045617 17098808 PMC1693934

[B37] Potterat O. (2010). Goji (*Lycium barbarum* and *L. chinense*): phytochemistry, pharmacology and safety in the perspective of traditional uses and recent popularity. Planta Med. 76, 7–19. 10.1055/s-0029-1186218 19844860

[B38] Schwarz D. S. Hutvágner G. Du T. Xu Z. Aronin N. Zamore P. D. (2003). Asymmetry in the assembly of the RNAi enzyme complex. Cell 115, 199–208. 10.1016/S0092-8674(03)00759-1 14567917

[B39] Sieber P. Wellmer F. Gheyselinck J. Riechmann J. L. Meyerowitz E. M. (2007). Redundancy and specialization among plant microRNAs: role of the miR164 family in developmental robustness. Development 134, 1051–1060. 10.1242/dev.02817 17287247

[B40] Storey J. D. Tibshirani R. (2003). Statistical significance for genomewide studies. Proc. Natl. Acad. Sci. U.S.A. 100, 9440–9445. 10.1073/pnas.1530509100 12883005 PMC170937

[B41] Turner M. Adhikari S. Subramanian S. (2013). Optimizing stem-loop qPCR assays through multiplexed cDNA synthesis of U6 and miRNAs. Plant Signal. Behav. 8:e24918. 10.4161/psb.24918 23673353 PMC4010539

[B42] Varkonyi-Gasic E. Hellens R. (2011). Quantitative stem-loop RT-PCR for detection of microRNAs, in RNAi and Plant Gene Function Analysis, eds Kodama H. Komamine A. (New York, NY: Humana Press), 145–157. 10.1007/978-1-61779-123-9_10 21533691

[B43] Wang L. Feng Z. Wang X. Wang X. Zhang X. (2010). DEGseq: an R package for identifying differentially expressed genes from RNA-seq data. Bioinformatics 26, 136–138. 10.1093/bioinformatics/btp612 19855105

[B44] Wang S. H. Liu J. K. Feng Y. Y. Niu X. L. Giovannoni J. Liu Y. S. (2008). Altered plastid levels and potential for improved fruit nutrient content by downregulation of the tomato DDB1-interacting protein CUL4. Plant J. 55, 89–103. 10.1111/j.1365-313X.2008.03489.x 18363785

[B45] Wen M. Shen Y. Shi S. H. Tang T. (2012). miREvo: an integrative microRNA evolutionary analysis platform for next-generation sequencing experiments. BMC Bioinformatics 13:140. 10.1186/1471-2105-13-140 22720726 PMC3410788

[B46] Wu H. J. Ma Y. K. Chen T. Wang M. Wang X. J. (2012). PsRobot: a web-based plant small RNA meta-analysis toolbox. Nucleic Acids Res. 40, W22–W28. 10.1093/nar/gks554 22693224 PMC3394341

[B47] Wu J. Wang D. Liu Y. Wang L. Qiao X. Zhang S. (2014). Identification of miRNAs involved in pear fruit development and quality. BMC Genomics 15:953. 10.1186/1471-2164-15-953 25366381 PMC4233070

[B48] Xie Z. Allen E. Fahlgren N. Calamar A. Givan S. A. Carrington J. C. (2005). Expression of Arabidopsis miRNA genes. Plant Physiol. 138, 2145–2154. 10.1104/pp.105.062943 16040653 PMC1183402

[B49] Xu Q. Liu Y. Zhu A. Wu X. Ye J. Yu K.. (2010). Discovery and comparative profiling of microRNAs in a sweet orange red-flesh mutant and its wild type. BMC Genomics 11:246. 10.1186/1471-2164-11-246 20398412 PMC2864249

[B50] Yang Z. Ebright Y. W. Yu B. Chen X. (2006). HEN1 recognizes 21-24 nt small RNA duplexes and deposits a methyl group onto the 2′ OH of the 3′ terminal nucleotide. Nucleic Acids Res. 34, 667–675. 10.1093/nar/gkj474 16449203 PMC1356533

[B51] Young M. Wakefield M. J. Smyth G. K. Oshlack A. (2010). Gene ontology analysis for RNA-seq: accounting for selection bias. Genome Biol. 11, R14. 10.1186/gb-2010-11-2-r14 20132535 PMC2872874

[B52] Zeng S. H. Liu Y. L. Wu M. Liu X. M. Shen X. F. Liu C. Z.. (2014). Identification and validation of reference genes for quantitative real-time PCR normalization and its applications in *Lycium*. PLoS ONE 9:e97039. 10.1371/journal.pone.0097039 24810586 PMC4014596

[B53] Zhou L. Chen J. Li Z. Li X. Hu X. Huang Y.. (2010). Integrated profiling of microRNAs and mRNAs: microRNAs located on Xq27.3 associate with clear cell renal cell carcinoma. PLoS ONE 5:e15224. 10.1371/journal.pone.0015224 21253009 PMC3013074

[B54] Zhu M. Chen G. Zhou S. Tu Y. Wang Y. Dong T.. (2014). A new tomato NAC (NAM/ATAF1/2/CUC2) transcription factor, SlNAC4, functions as a positive regulator of fruit ripening and carotenoid accumulation. Plant Cell Physiol. 55, 119–135. 10.1093/pcp/pct162 24265273

