# Supplementary figures and images for: Identification and characterization of miRNAs in ripening fruit of Lycium barbarum L. using high-throughput sequencing
Source: Front Plant Sci. 2015 Sep 25;6:778. doi: 10.3389/fpls.2015.00778 (PMC4585183; doi:10.3389/fpls.2015.00778)

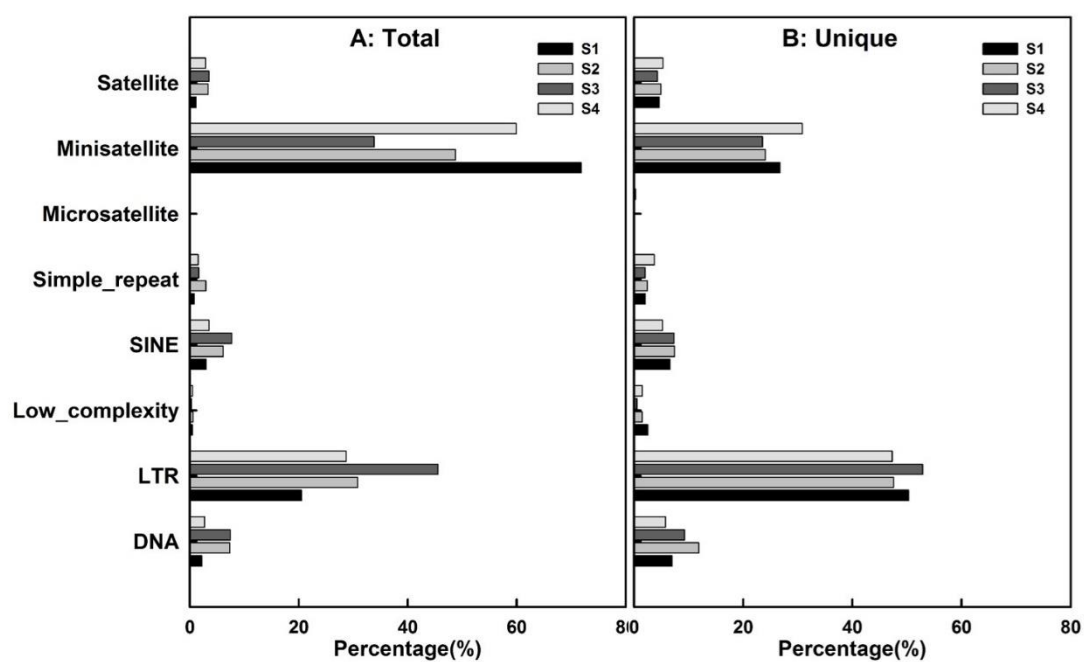

Figure S1

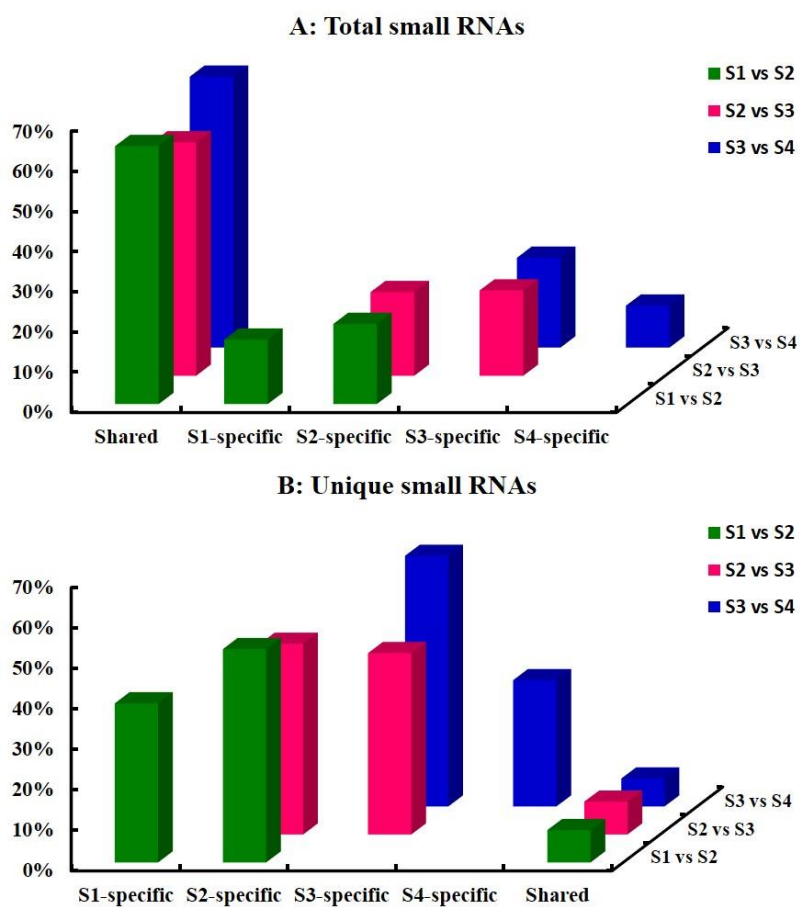

Figure S2

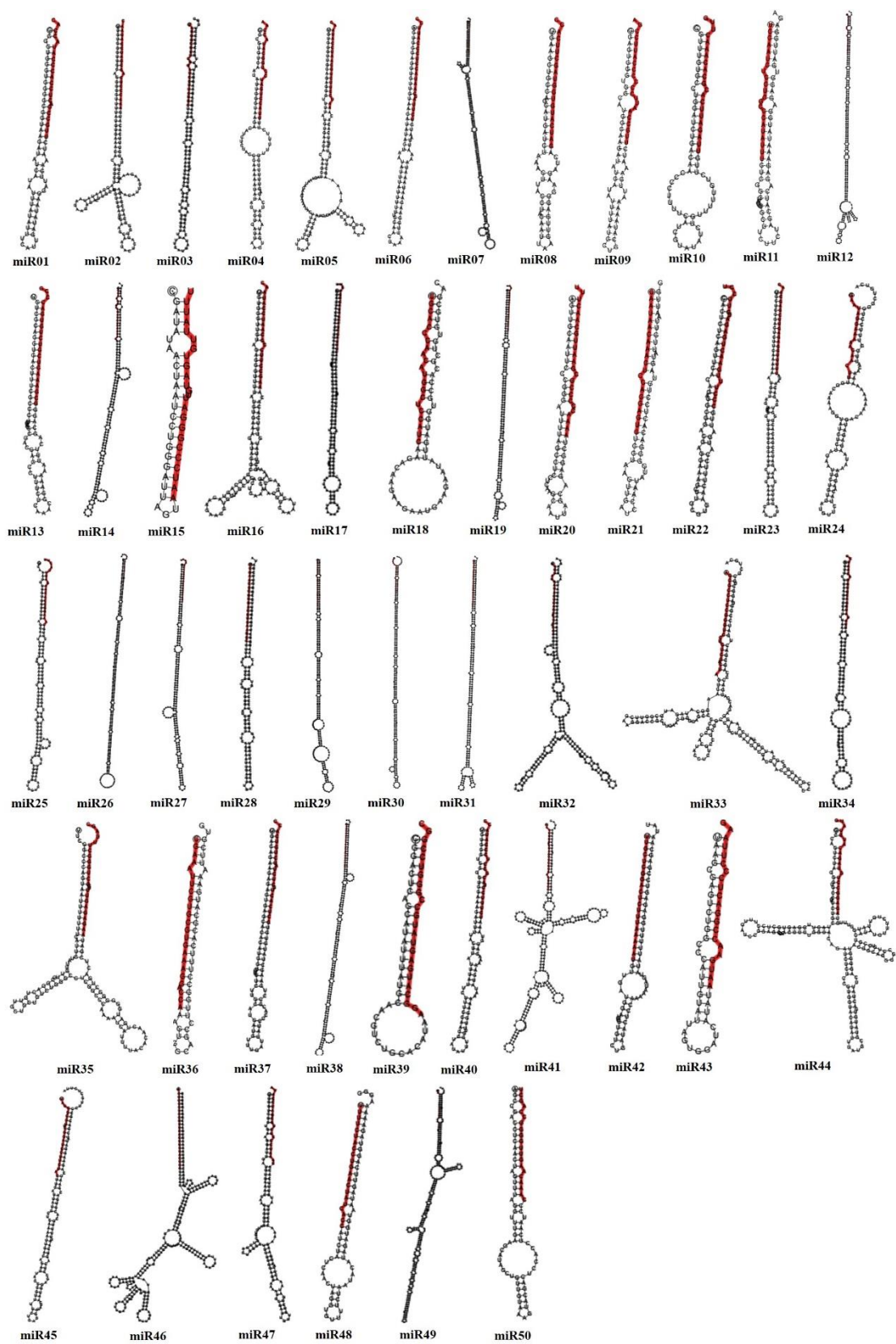

**Figure S3**

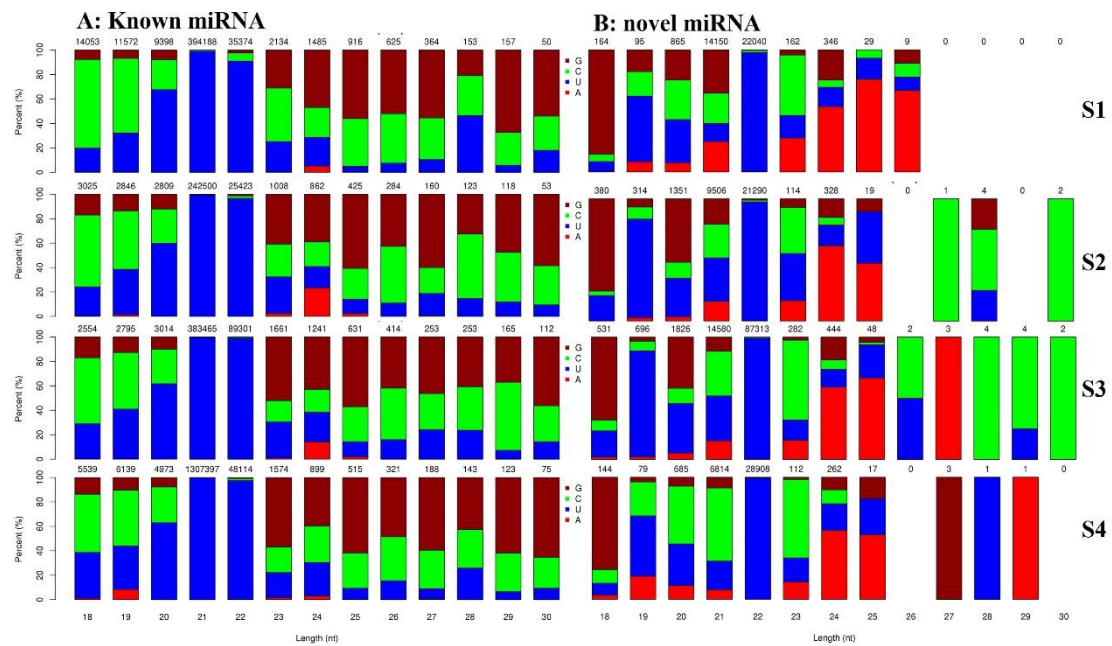

Figure S4

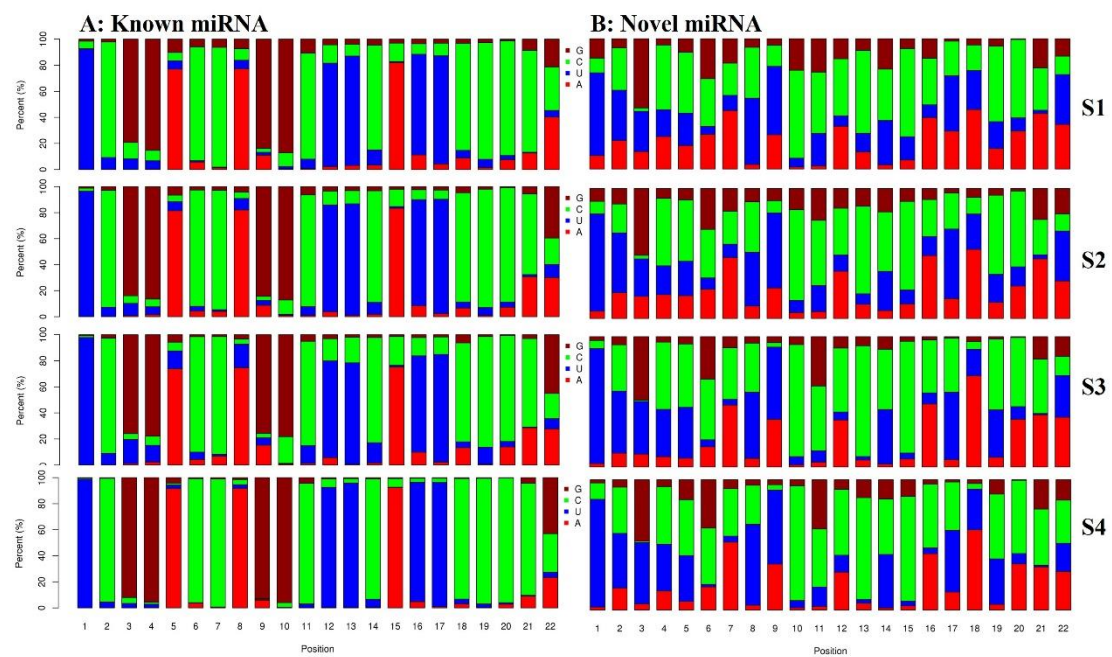

Figure S5

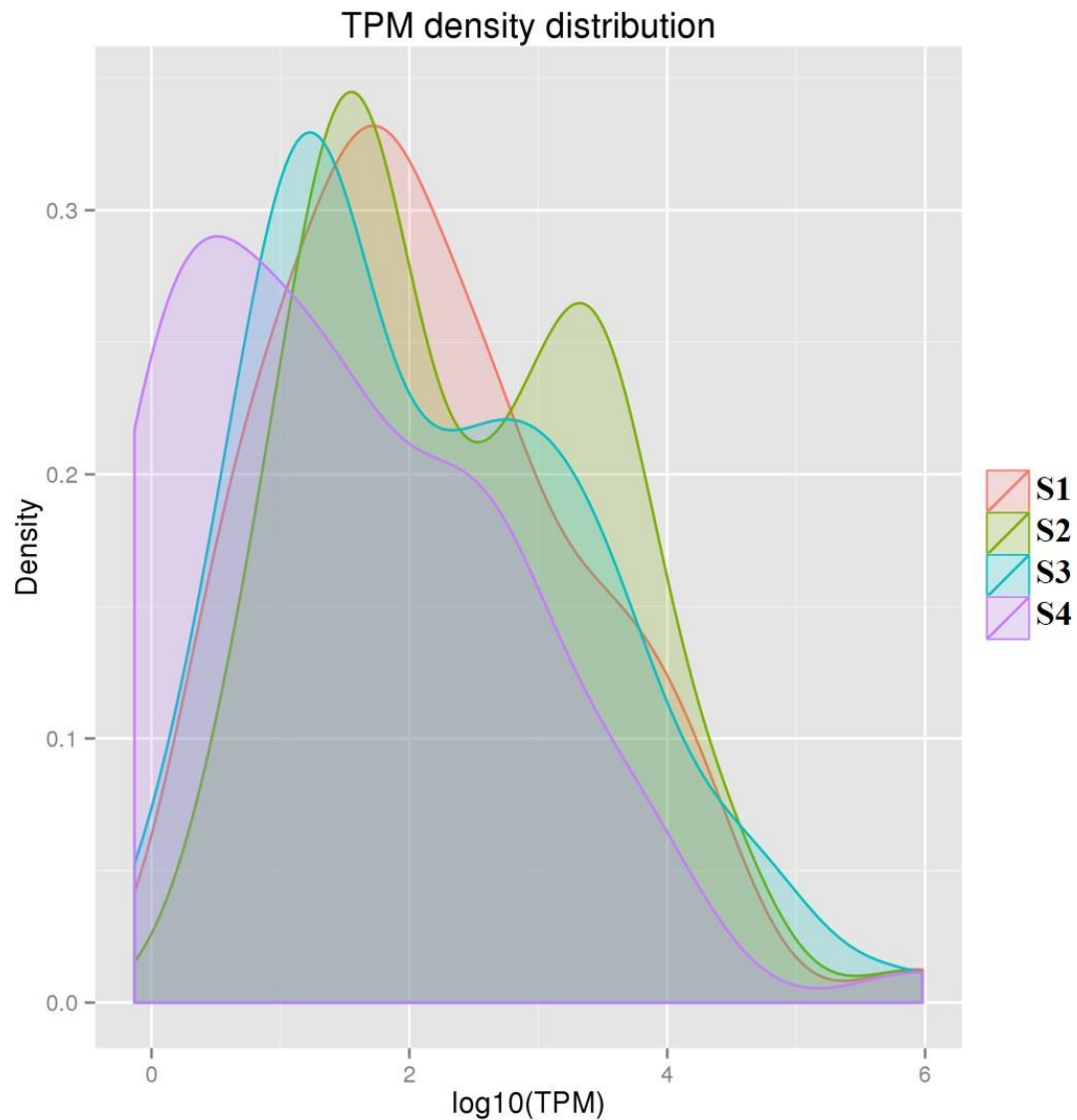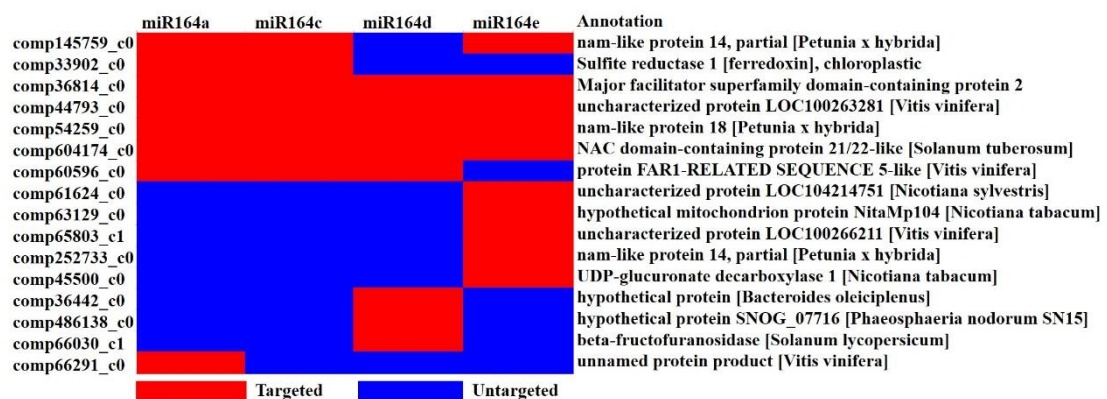

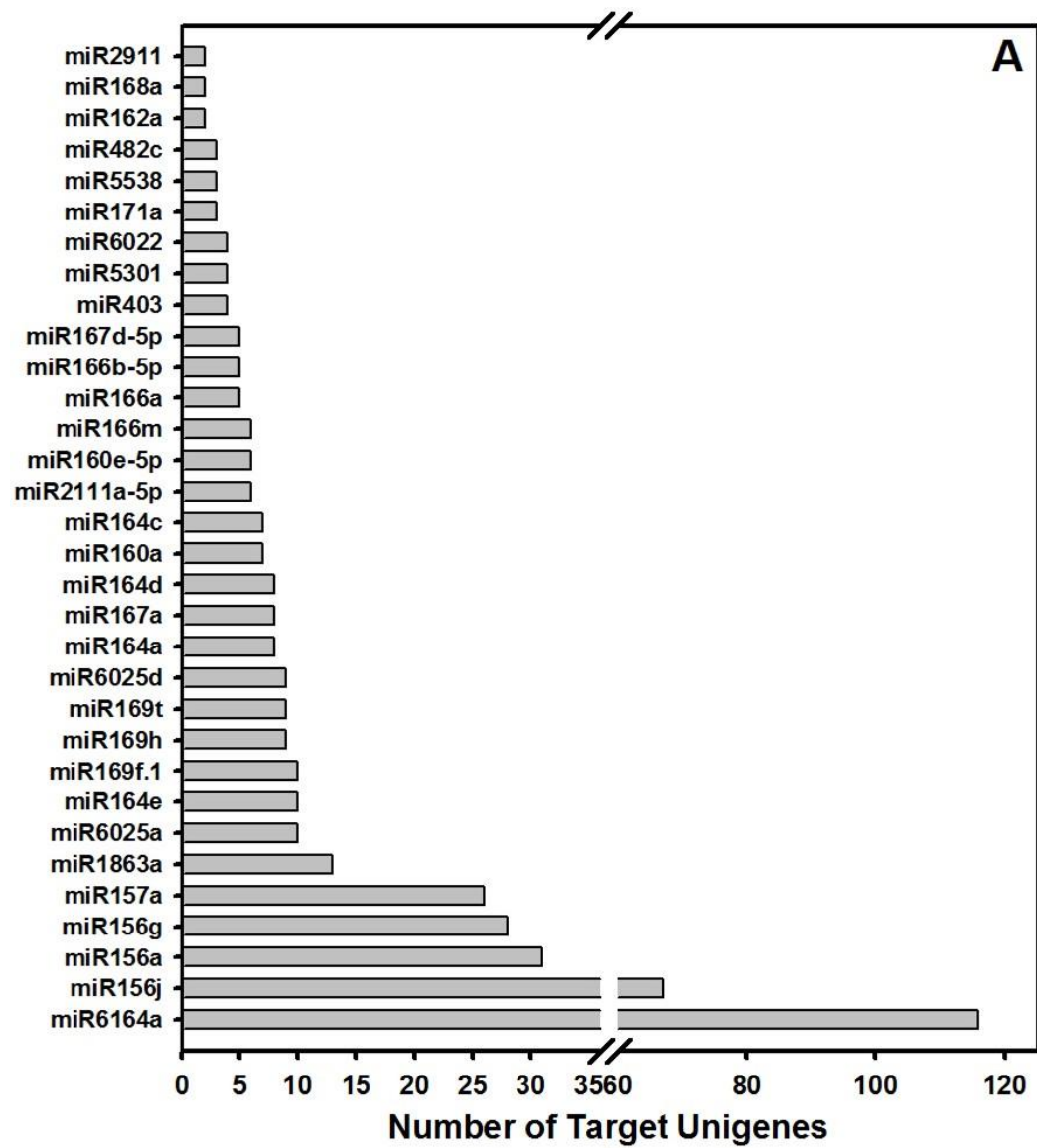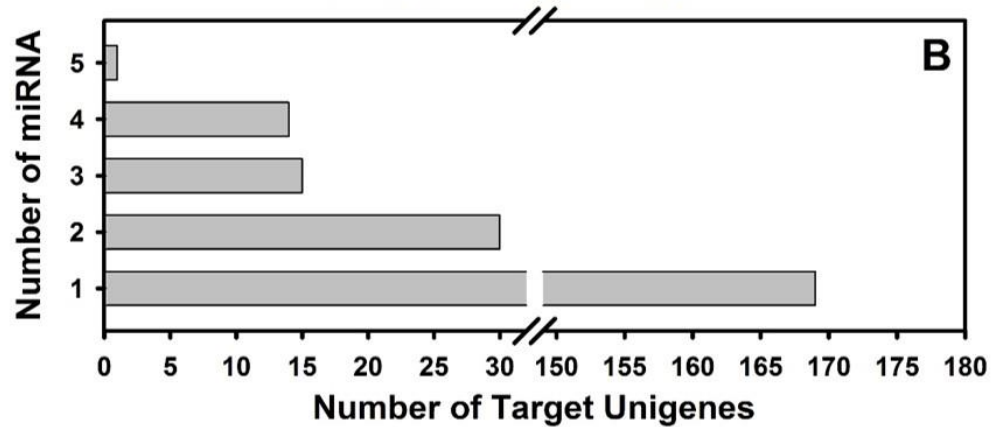

Figure S8

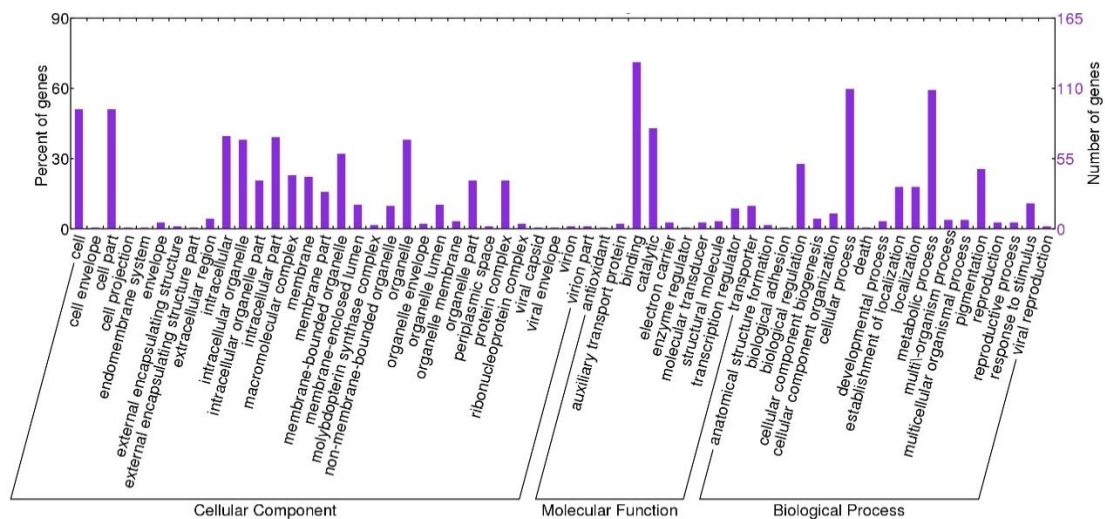

Figure S9

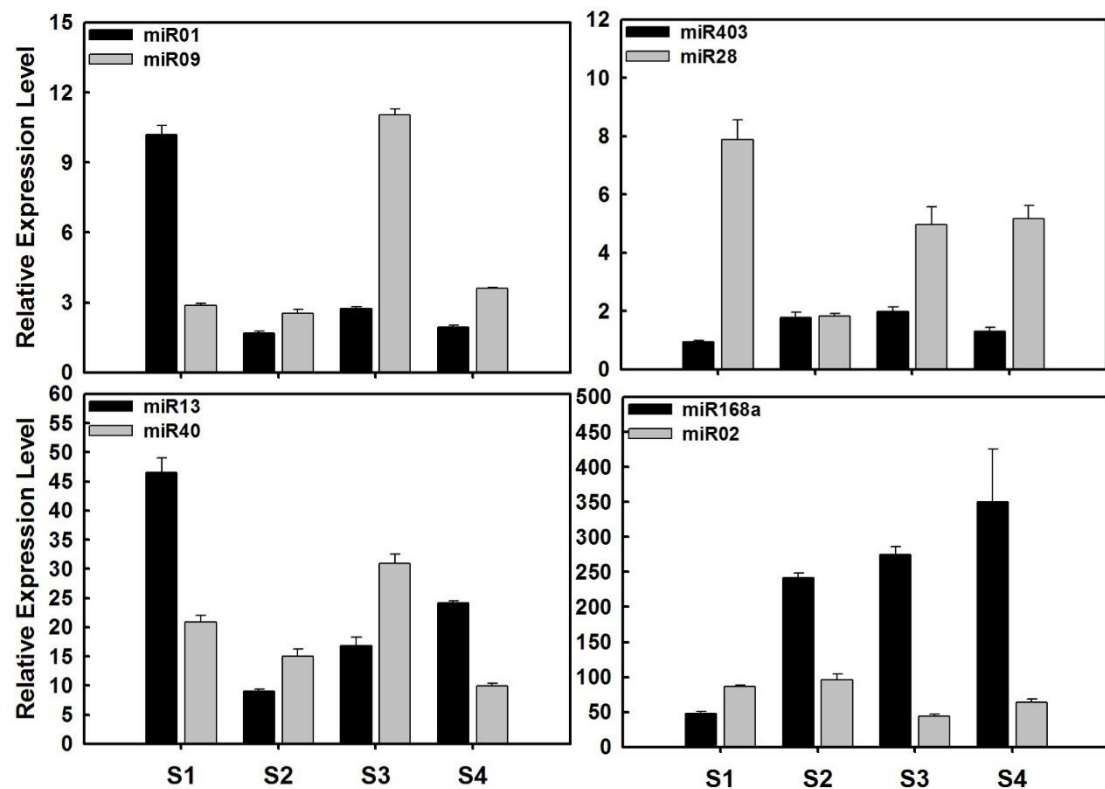

Figure S10

Supplement: Figure S1 — Repeat classification of total (A) and unique (B) sRNA reads. [file Image1.PDF]
